# Rapamycin and Low-dose IL-2 Mediate an Immunosuppressive Microenvironment to Inhibit Benign Prostatic Hyperplasia

**DOI:** 10.7150/ijbs.85089

**Published:** 2023-07-03

**Authors:** Tianyu Cao, Feng Xie, Youwei Shi, Junhao Xu, Yi Liu, Di Cui, Fang Zhang, Lihui Lin, Weize Li, Yanting Gao, Yuan Ruan, Xiaohai Wang, Yiping Zhu, Bangmin Han, Shujie Xia, Wenhuan Guo, Bin Li, Yifeng Jing

**Affiliations:** 1 Department of Urology, Shanghai General Hospital, Shanghai Jiao Tong University School of Medicine, Shanghai, China.; 2 Department of Immunology and Microbiology, Shanghai Institute of Immunology, Shanghai Jiao Tong University School of Medicine, Shanghai, China.; 3 Department of Plastic Surgery, Shanghai General Hospital, Shanghai Jiao Tong University School of Medicine, Shanghai, China.; 4 Department of Laboratory Medicine, Shanghai General Hospital, Shanghai Jiao Tong University School of Medicine, Shanghai, China.; 5 Department of Pathology, Shanghai Ninth People's Hospital, Shanghai Jiao Tong University School of Medicine, Shanghai, China.; 6 Center for Immune-Related Diseases at Shanghai Institute of Immunology, Department of Respiratory and Critical Care Medicine of Ruijin Hospital, Department of Immunology and Microbiology, Shanghai Jiao Tong University School of Medicine, Shanghai, China.; 7 Department of Thoracic Surgery, Clinical Translational Research Center, Shanghai Pulmonary Hospital, Tongji University School of Medicine, Shanghai, China.; 8 Institute of Arthritis Research, Guanghua Integrative Medicine Hospital, Shanghai University of Traditional Chinese Medicine, Shanghai, China.; 9 Department of Integrated TCM & Western Medicine, Shanghai Skin Disease Hospital, School of Medicine, Tongji University, Shanghai, China.; 10 Henan Key Laboratory for Digestive Organ Transplantation, Department of Hepatobiliary and Pancreatic Surgery, The First Affiliated Hospital of Zhengzhou University, Zhengzhou, China.; 11 Shenzhen Key Laboratory of Immunity and Inflammatory Diseases, Shenzhen, China.

**Keywords:** BPH, Immune microenvironment, Treg

## Abstract

Benign prostatic hyperplasia (BPH) is a condition that becomes more common with age and manifests itself primarily as the expansion of the prostate and surrounding tissues. However, to date, the etiology of BPH remains unclear. In this respect, we performed single-cell RNA sequencing of prostate transition zone tissues from elderly individuals with different prostate volumes to reveal their distinct tissue microenvironment. Ultimately, we demonstrated that a reduced Treg/CD4+ T-cell ratio in the large-volume prostate and a relatively activated immune microenvironment were present, characterized partially by increased expression levels of granzymes, which may promote vascular growth and profibrotic processes and further exacerbate BPH progression. Consistently, we observed that the prostate gland of patients taking immunosuppressive drugs usually remained at a smaller volume. Furthermore, in mouse models, we confirmed that both suppression of the immune system with rapamycin and induction of Treg proliferation with low doses of IL-2 therapy indeed prevented the progression of BPH. Taken together, our findings suggest that an activated immune microenvironment is necessary for prostate volume growth and that Tregs can reverse this immune activation state, thereby inhibiting the progression of BPH.

## Background

Benign prostatic hyperplasia (BPH) is the most common cause of lower urinary tract symptoms (LUTS) in elderly men due to bladder outlet obstruction. The incidence of histologic BPH is greater than 50% in the male population over the age of 60 years, and it goes up to 83% by the age of 80 years 1. Despite many years of scientific research, the onset and constant progression mechanism of BPH remain to be elucidated. Prostate volume is one of the important risk factors for the progression of BPH, and the risk of acute urinary retention and the need for surgical intervention in patients with BPH increases significantly with increasing prostate volume 2-3. Interestingly, although old age is one of the necessary conditions for the development of BPH, the prostate does not continue to grow in all elderly men. Prostates that grow continuously by more than 80 ml are defined as large prostates, while those that do not have significant enlargement and continue to maintain a volume of no more than 30 ml are defined as small prostates 4. Testosterone plays a crucial role in the development of the prostate gland in the fetal and adolescent stages. However, in elderly individuals, the prostate undergoes a resurgence of growth despite a decline in serum testosterone levels 5. The mechanisms underlying this significant difference in prostate growth characteristics due to age and testosterone are not clear but are worth exploring.

Currently, chronic inflammation is considered the main contributor to the onset and progression of BPH 6. The immune cell proinflammatory microenvironment has been linked to BPH, but the precise relationship between the immune status of the prostate and BPH, particularly its correlation with clinical symptoms such as LUTS, remains largely unclear 7-8. T lymphocytes are an essential component of the adaptive immune system, acting as mediators and effectors in the immune system 9-11. One of the characteristics of prostate tissue chronic inflammation is the accumulation of immune cells, especially T lymphocytes. In particular, studies have shown that CD8+ T lymphocytes are the most abundant lymphocyte population around the prostate 12. Regulatory T cells (Tregs) are a specialized phenotypic population of CD4+ T cells involved in multiple diseases, such as cancer, fibrosis, infection, and autoimmune diseases (AID) 13-15. Tregs participate in shaping the immune microenvironment primarily by performing immunosuppressive functions to prevent autoimmune and tissue damage caused by excessive or unnecessary immune activation. Several therapies targeting immunosuppression have been reported to be effective in multiple diseases. Rapamycin, a widely utilized inhibitor of the mammalian target of rapamycin (mTOR) pathway, has been demonstrated to exert crucial functions in preventing posttransplant rejection and antitumor and antiaging processes. Its mechanism involves hindering the functional activation of dendritic cells, restraining the proliferation of effector T cells, and facilitating the differentiation of Tregs, ultimately leading to the suppression of immune system activation 16. Low-dose IL-2 can promote the proliferation of Tregs and play a reliable role in clinical trials of AID 14. However, the specific effectors of T-cell involvement in the BPH process remain unclear, and whether the progression of BPH can be restrained by Treg-enhancing therapies to effectively suppress the inflammatory status still needs further investigation.

In this study, we investigated the correlation between the level of immune activation in the prostate microenvironment and BPH. We performed single-cell RNA sequencing (scRNA-seq) of the prostate transition zone to investigate the immune status difference between large and small prostates. Our findings revealed that the immune microenvironment in the large prostate was relatively activated with an abundance of granzymes and had less Treg infiltration than that in the small prostate. We also observed that kidney transplant patients receiving immunosuppressive agents had smaller prostate volumes and did not experience age-related increases in prostate volume. In addition, animal experiments were conducted to demonstrate that rapamycin or low-dose IL-2 treatment could alleviate testosterone- induced BPH in mice. Patient medical records and human prostate tissue observations both support the repurposing of approved therapeutics. Together, our results provide a detailed characterization of the immune microenvironment in different volumes of the prostate and further reveal that immunosuppressants or low-dose IL-2-induced Treg- enhanced therapy can effectively inhibit prostate hyperplasia in mice, thus providing new insights for clinical BPH treatment.

## Methods

### Tissue disaggregation of human tissue

The acquisition of human prostate tissue was approved by the Ethics Committee of Shanghai General Hospital after obtaining informed consent from the patients. Large prostate tissue was obtained from a male patient receiving thulium laser enucleation of the prostate (ThuLEP) for BPH, whose preoperative transabdominal B-ultrasound prostate volume was greater than 80 mL. Small prostate tissue was obtained from a male patient undergoing ThuLEP due to bladder neck contracture, whose preoperative transabdominal B-ultrasound prostate volume was less than 30 mL. All patients were older than 70 years old, and had not received any BPH- related treatment before operation, and postoperative pathology showed no prostate cancer.

Tissue for scRNA-seq were minced and digested with 1000 U/mL Collagenase I (Biosharp) + 1 mg/mL DNAse I (Absin) + 1% antibiotic/antimycotic for 4 h while shaking at 37 °C, followed by treatment with TrypLE Express (Gibco) reagent as above. After cell washing and RBC lysis, cell suspensions were subjected to debris removal with Debris Removal Solution (Miltenyi Biotec).

Tissues for flow cytometry were minced, then digested while shaking at 37 °C for 2 h in 200 U/mL Collagenase I + 1 mg/mL DNase I + 1% antibiotic/antimycotic solution in Hank's Balanced Salt Solution (Beyotime). Digestion solution was replaced with TrypLE Express dissociation reagent and allowed to shake at 37 °C for 5-10 min. Digested samples were neutralized in DMEM (Gibco) + 10% FBS (Gibco), then mechanically disrupted by pipetting repeatedly. Samples were passed through a 100 µm cell strainer, then washed. Red blood cells were lysed in a hypotonic buffer.

### scRNA-seq of prostatic cells

10X Chromium Chip Single-cell library generation and preparation were performed on the single-cell suspension according to 10X Chromium 3′ solution (V2 kit) as per manufacturer's instructions with an aim to capture 5000-10000 cells/channel. Sequencing was performed on the Illumina HiSeq4000 platform. Following sequencing BCL files were demutiplexed to Fastq files using CASAVA.

### Raw data processing and quality control

The Cell Ranger version 5.0.0 software pipeline provided by 10×Genomics was used to demultiplex cellular barcodes, map reads to the genome and transcriptome using the STAR aligner, and down-sample reads as required to generate normalized aggregate data across samples, producing a matrix of gene counts versus cells.

R version 4.1.1 and Python version 3.9 were used in all statistical analyses. Cells that had fewer than 500 observed genes were discarded. Cells were also removed if greater than 20% of all reads mapped to mitochondrial genes.

### Unsupervised clustering and identification

Seurat version 4.1 was used for data normalization and cell clustering based on differential gene expression. All cells' data were normalized using BBKNN version 1.3.3. These corrected data, after permutation and selection of the first 50 principal components based on principal component analysis (PCA) scores, were used for downstream analysis. Unsupervised clustering was performed in Seurat, which uses graph-based approaches to first construct K-nearest neighbor graphs (K = 10) and identifies clusters by iteratively forming communities of cells to optimize the modularity function. The number of clusters were determined using the Louvain algorithm for community detection, as implemented in Seurat with a resolution of 0.4. Differentially expressed genes (DEGs) were selected using the FindMarkers function (test.use = presto) in Seurat. P value < 0.05 and |log2foldchange| > 0.25 was set as the threshold for significantly differential expression. GO enrichment and KEGG pathway enrichment analysis of DEGs were respectively performed using R based on the hypergeometric distribution.

### Gene Set Enrichment Analysis (GSEA)

GSEA was used to complete GO and KEGG term enrichment analysis with the Molecular Signatures Database (MSigDB) C5 GO gene sets and C2 KEGG gene sets Version 7.2 separately.

### scMetabolism Analysis

scMetabolism version 0.2.1 was performed to quantify the metabolic activity with single- cell sequencing data. Specifically, we scored the metabolic pathway activity in each cell using VISION algorithm. scMetabolism preseted metabolic gene sets, including 85 KEGG pathways and 82 REACTOME pathways.

### CellChat Analysis

The cell communication analysis was performed using the CellChat version 1.1.3 R package. First, we imported the normalized expression matrix to create the cellchat object with the createCellChat function. Secondly, the data was preprocessed with the identifyOverExpressedGenes, identifyOverExpressedInteractions and projectData function using the default parameters. The computeCommunProb, filterCommunication (min.cells = 10) and computeCommunProbPathway functions were then used to determine any potential ligand-receptor interactions. Finally, the cell communication network was aggregated using the aggregateNet function.

### Immunohistochemistry

Prostate sections were deparaffinized, treated with heat-induced epitope retrieval (HIER) and incubated with primary antibodies against GZMB (1:3000, Abcom), GZMK (1:1000, Abcom for human; 1:8000, Affinity for mouse), GZMA (1:2000 for human, 1:4000 for mouse, Proteintech), CD31 (1:200, Abcom) for 1h at room temperature. Then secondary antibody (GTvision, GK5005) was applied for 30 minutes at room temperature. The staining was developed using Diaminobenzidine and tissues were counterstained using hematoxylin then. The staining of samples was evaluated by pathologist independently. All human and mouse IHC quantitation was blindly completed by counting the indicated number of fields under the 40x objective.

### Immunofluorescence

Prostate sections were deparaffinized, treated with HIER incubated with primary antibodies against CD4 (1:200 for Human, 1:1000 for mouse, Abcom), Foxp3 (1:400 for Human, 1:500 for mouse, Abcom). Staining was completed using the Fluorescence immunohistochemistry kit (Panovue), following the manufacturer's instructions. The staining of samples was evaluated by pathologist independently. All human and mouse quantitation was blindly completed by counting the indicated number of fields under the 40x objective.

### Prostate cell flow cytometry

The prostate single cell suspension was centrifuged, and the supernatant was removed, and 50uL normal saline was added to resuspend. Add CD45-KO, CD3-FITC, CD4-ECD, CD8-PC5, CD25-PC7, CD127-APC flow antibodies (both 2μL, Beckman Coulter) to the cell suspension and incubate at room temperature for 20 minutes. After incubation, centrifuge to remove the supernatant, add 50uL saline to resuspend, and use a Beckman Navios flow cytometer. The results were analyzed using Kaluza 3.13 flow cytometry analysis software.

### Animal studies

All animal experiments were performed under the approval of IACUC of Shanghai General Hospital. Male C57B6/L mice on the inbred were purchased from The Cyagen Bioscience Inc. (Guangzhou, China). All mice were maintained under specific-pathogen-free (SPF) conditions. 8-week-old mice were used in all experiments. The 8-week-old mice in the experimental group were castrated after being anesthetized with isoflurane. One week later, they were subcutaneously injected with 5mg/kg testosterone propionate (MCE) for 30 consecutive days to establish BPH model. Mice in different experimental groups were intragastrically administered with 1 mg/kg rapamycin or intraperitoneally injected with low- dose IL-2 at 25 μg/kg as planned (on days 1-5, 9, 13, 17, 21, 25, 29, 30). Euthanize the mice with CO2, weigh the mice, and remove the mouse bladder and part of the urethra. Mouse prostates were isolated using a Stereo microscope, weighed and fixed with 4% paraformaldehyde. Mouse prostate index = mouse prostate weight (mg) / mouse body weight (g).

### Prostate volume data acquisition

100 men over 50 years old who underwent transabdominal prostate B-ultrasound examination at the Medical Physical Examination Center of Shanghai First People's Hospital in November 2022, and 67 men over 50 years old who underwent transabdominal prostate B-ultrasound examination from September 2021 to November 2022 male kidney transplant patients were included in the study. Medication data of kidney transplant patients were derived from their medical records. The calculation method of prostate volume is prostate length*width*height*0.52.

### Statistical analysis

Data are presented as mean ± SEM. Differences between groups were analyzed with Student's t-test or Chi-square test according to different sample types. Statistical analyses were performed using SPSS 27.0 and Graphpad Prism 9.4. A p-value of less than 0.05 was considered significant. In data figures, significance is indicated by *p < 0.05, **p < 0.01, ***p < 0.001, and ****p < 0.0001.

## Results

### scRNA-seq of the prostate transition zone reveals the difference in the cellular composition of large and small prostates

**Figure 1:**
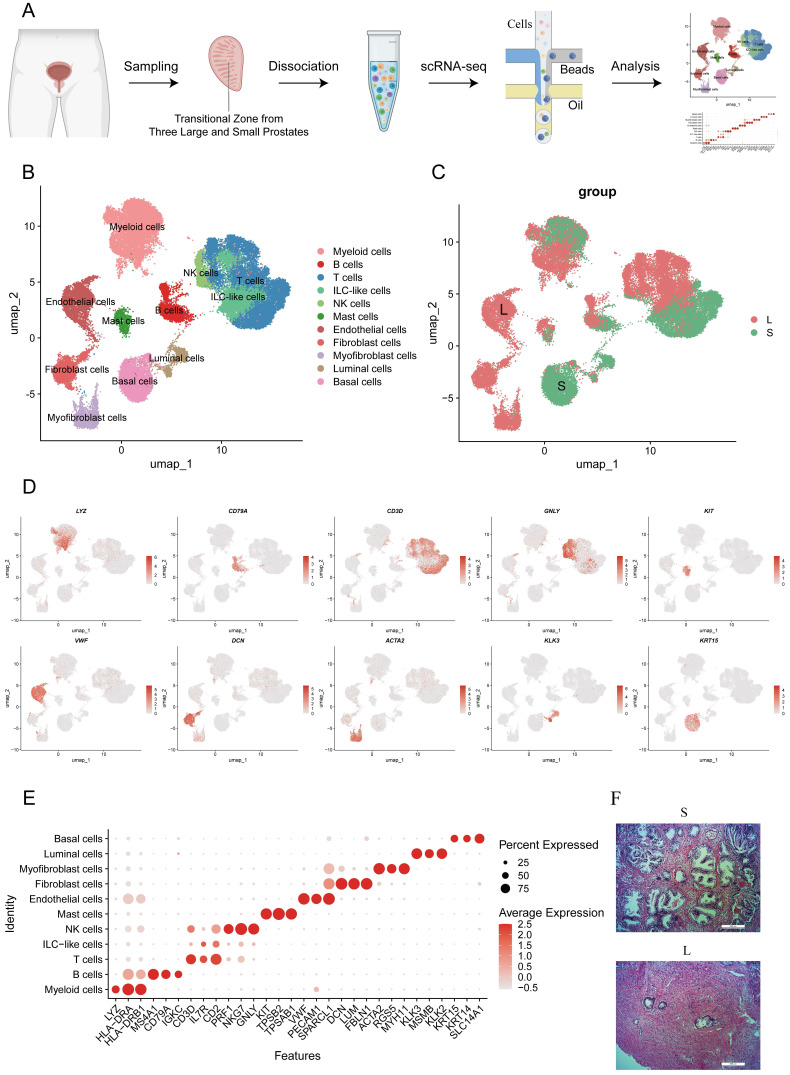
** Identification of human prostate cell clusters with Single-Cell RNA Sequencing. (A)** Schematic of human prostate transition zone tissue collection and processing for single-cell RNA sequencing. **(B)** Aggregated scRNA-seq data from ten prostate specimens with subclustering into myeloid, B, T, ILC-like, NK, mast, endothelial, fibroblast, myofibroblast, luminal and basal cells. Clusters were identified and re-merged. **(C) C**ells are divided according to their prostate size origin.** (D)** Specific gene plot of each cell subpopulation. **(E)** Dot plot of cluster-specific genes after in silico supervised identification of prostate. **(F)** HE-stained histopathological sections of large prostate and small prostates.

Human prostate transition zone tissues were excised from three large prostates and three small prostates separately without any BPH drug treatment, and then they were dissociated and subjected to scRNA-seq. We integrated expression profiles across all patients and obtained a total of 44,566 cells, with approximately 8000 cells per patient, after quality control and batch effect removal, resulting in a comprehensive atlas of human prostate transition zone tissues (Fig. 1A, S1A). Based on classical marker genes, the scRNA-seq profiles were then annotated into 10 cell lineages (Fig. 1B-D). The cell composition of large and small prostates was mostly different (Fig. 1E, S1B-C). Similar to what we observed histologically (Fig. 1F), the large prostates had a higher proportion of stromal cells, while the proportion of gland cells was higher in small prostates. Although the large and small prostates contained similar proportions of the same type of cells, such as T, B, NK and other immune cells, their positions in the graphs were dramatically different, which indicated that large and small prostates differed not only in histology but also in their cellular functions.

### Large and small prostates are composed of different T-cell subsets

**Figure 2:**
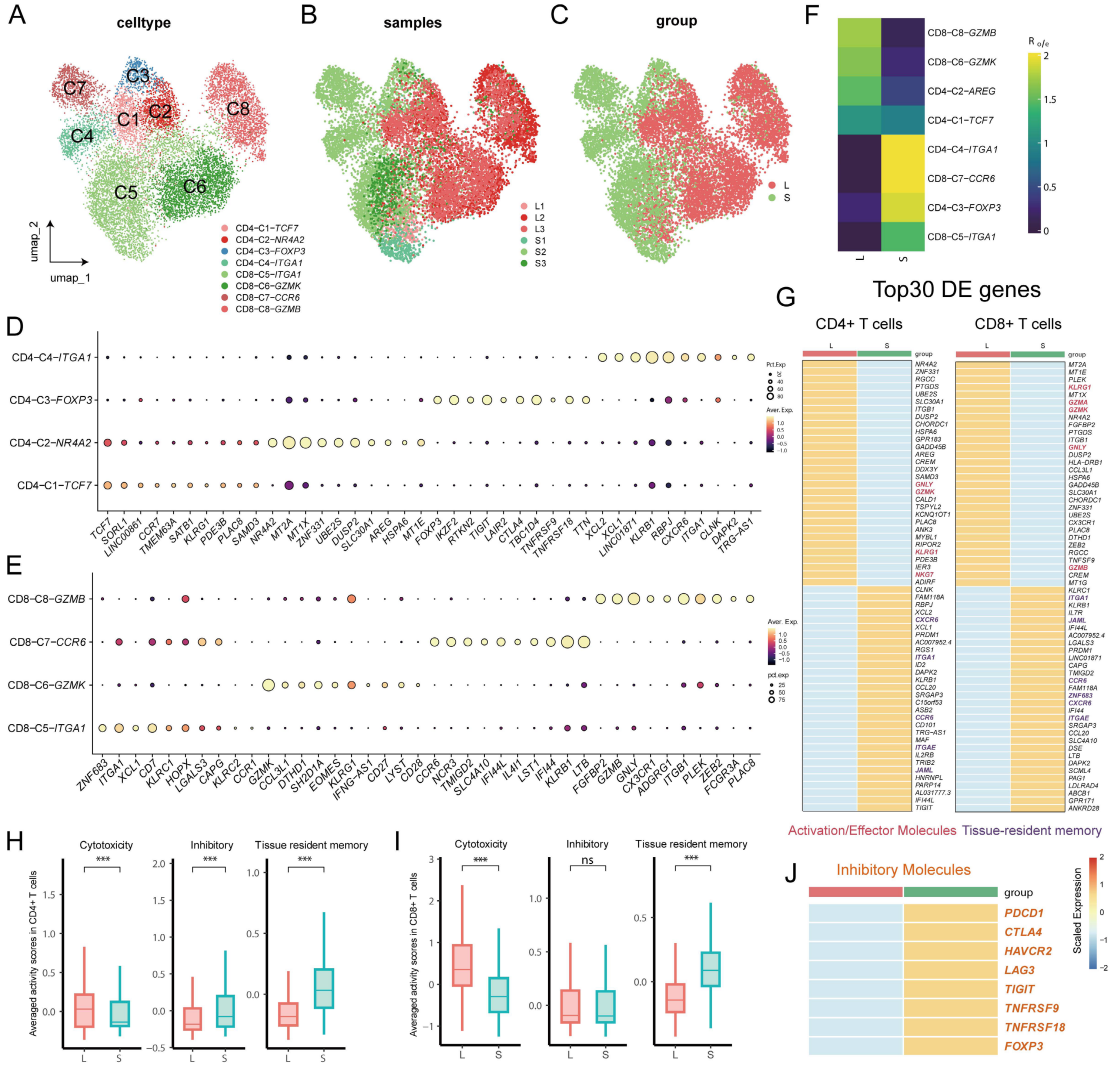
** Identification of T cell clusters in prostates. (A)** T cells were divided into 8 subclusters according to unsupervised cluster analysis. **(B-C)** T cells are divided according to their original prostatic size and identities. **(D-E)** Dot plot of cluster- specific genes after in silico supervised identification of CD4+ T cells and CD8+ T cells. **(F)** OR analysis of the T cell clusters in large and small prostates. **(G)** Differentially expressed genes of CD4+ T and CD8+ T cells in large and small prostates. **(H-I)** Averaged activity scores for the indicated gene signatures of CD4+ T cells and CD8+ T cells in large and small prostates. **(J)** Inhibitory genes expression on CD4+ T cells in large and small prostates. (ns p≥0.05; *** p≤0.001).

Infiltration of immune cells, especially T cells, and secretion of proinflammatory mediators have been shown to be involved in the pathogenesis of BPH 17. Therefore, we reclustered T cells for downstream analysis to reveal the characteristics of the immune system in the development of BPH. The reclustering of T cells revealed 8 clusters, which were designated CD8+ T cells (CD8A^+^) and CD4+ T cells (CD4^+^) (Fig. 2A, S2A). CD4+ T cells were composed of *CCR7^+^* naive CD4 T cells (CD4-C1), *FOXP3^+^* Treg cells (CD4-C3), *ITGA1^+^* memory CD4+ T cells (CD4-C4) and *AREG^+^* CD4+ T cells enriched in metallothionein genes (CD4-C2) (Fig. 2D). CD8+ T cells were further divided into four subpopulations, with CD8-C6 and CD8-C8 expressing the effector genes *GZMK* and *GZMB*, respectively, while cells in CD8-C5 and CD8-C7 expressed the tissue-resident memory cell hallmark genes *ITGA1* and *ZNF683* versus *CXCR6* (Fig. 2E) 18. Among them, cells in the memory T-cell CD8-C7-*CCR6* and CD4-C4-*ITGA1* clusters both displayed a T_H_17 phenotype with upregulated expression of genes (*RORC*, *CCR6* and *KLRB1*) Fig. S2B) (19. Interestingly, we noted that even after batch effect removal, the distribution between T-cell subpopulations remained different between large and small prostates but with less heterogeneity within similar volumes (Fig. 2B-C), implying distinct immune homeostasis in large and small prostates. Odds ratio (OR) analysis further revealed that resident memory cells (CD4-C4-*ITGA1*, CD8-C7-*CCR6* and CD8-C5-*ITGA1*) and Treg cells appeared to be more abundant in small prostates, whereas effector CD8+ T cells (both CD8-C6-*GZMK* and CD8-C8-*GZMB)* showed a strong distribution preference in large prostates (Fig. 2F). Differentially expressed genes indicated higher expression of effector genes (such as *GZMK*, *GNLY, GZ*MA and *GZMB*) and lower resident memory gene expression (such as *ITGA1*, *CCR6*,* ITGAE*, *JAML* and *ZNF683*) in both CD4+ T and CD8+ T cells from the large prostate (Fig. 2G). However, there was no difference in *PRF1* expression of T cells between large and small prostate Fig. S2C). Comparison of averaged activity scores for the indicated gene signatures further confirmed similar features of T cells from large and small prostates (Fig. 2H and Fig. 2I). Moreover, CD4+ T cells from the small prostate exhibited higher expression of inhibitory receptor genes (such as *CTLA4*, *TIGIT*, *TNFRSF18* and *TNFRSF9*) (Fig. 2H, 2J), which were mainly expressed in Treg cells (Fig. 2D). Together, the above results suggested that both CD4+ T and CD8+ T cells in large prostates were more immune active, while CD4+ T cells in smaller prostates showed a potent immunosuppressive profile (9.

### Large prostates contain fewer Tregs

**Figure 3:**
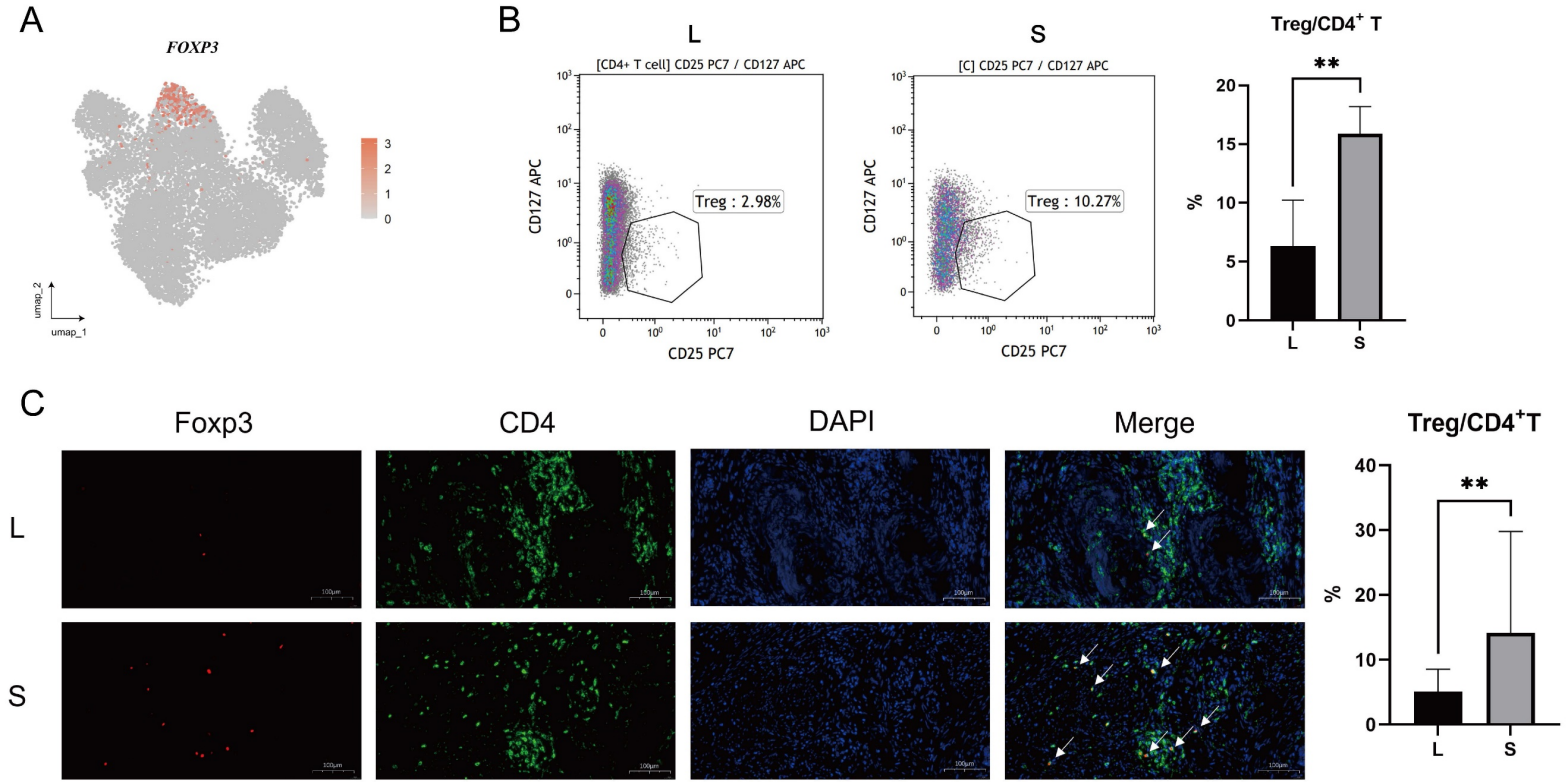
** Differences in the expression of Treg in the large and small prostates. (A)** FOXP3 expression plot in T cells of prostate. **(B)** Standard flow cytometry strategy for detection of Tregs in the prostate and statistical bar graph. **(C)** Immunofluorescence of large and small prostates displays different enrichment of Tregs in the transition zones. Right bars graph indicates statistical results of immunofluorescence. (** p≤0.01).

Tregs are immunosuppressive cells that are able to promote the formation of an immunosuppressive microenvironment 14-15. As mentioned above, CD4+ T cells from small prostates possess more immunosuppressive characteristics, and the associated inhibitory genes were mainly enriched in Treg cells (Fig 2J, 3A and S2D). Flow cytometry of prostatic transitional zone tissue confirmed a greater CD4+CD25+CD127lowTreg/CD4+T ratio in small prostates than in large prostates (Fig. 3B, S3A) (n = 7 in each group). Immunofluorescence further confirmed that small prostates contained more CD4 and Foxp3 coexpressing cells and that the CD4^+^Foxp3^+^T/CD4^+^T ratio was greater in small prostates than in large prostates (Fig. 3C) (n = 10 in each group). However, flow cytometric analysis showed no significant difference in the CD4+CD25+CD127lowT/CD4+T ratio between the peripheral blood of large and small prostate patients Fig. S3B, Table S1 (n = 71 in the large group, n = 52 in the small group). Unlike the previously reported higher percentage of Tregs in the peripheral blood of patients with small volume prostate 20, our data suggest that the difference in the proportion of Tregs in patients with a large or small prostate is confined within the prostate tissue, independent of peripheral blood.

### T cells are activated in large prostates

**Figure 4:**
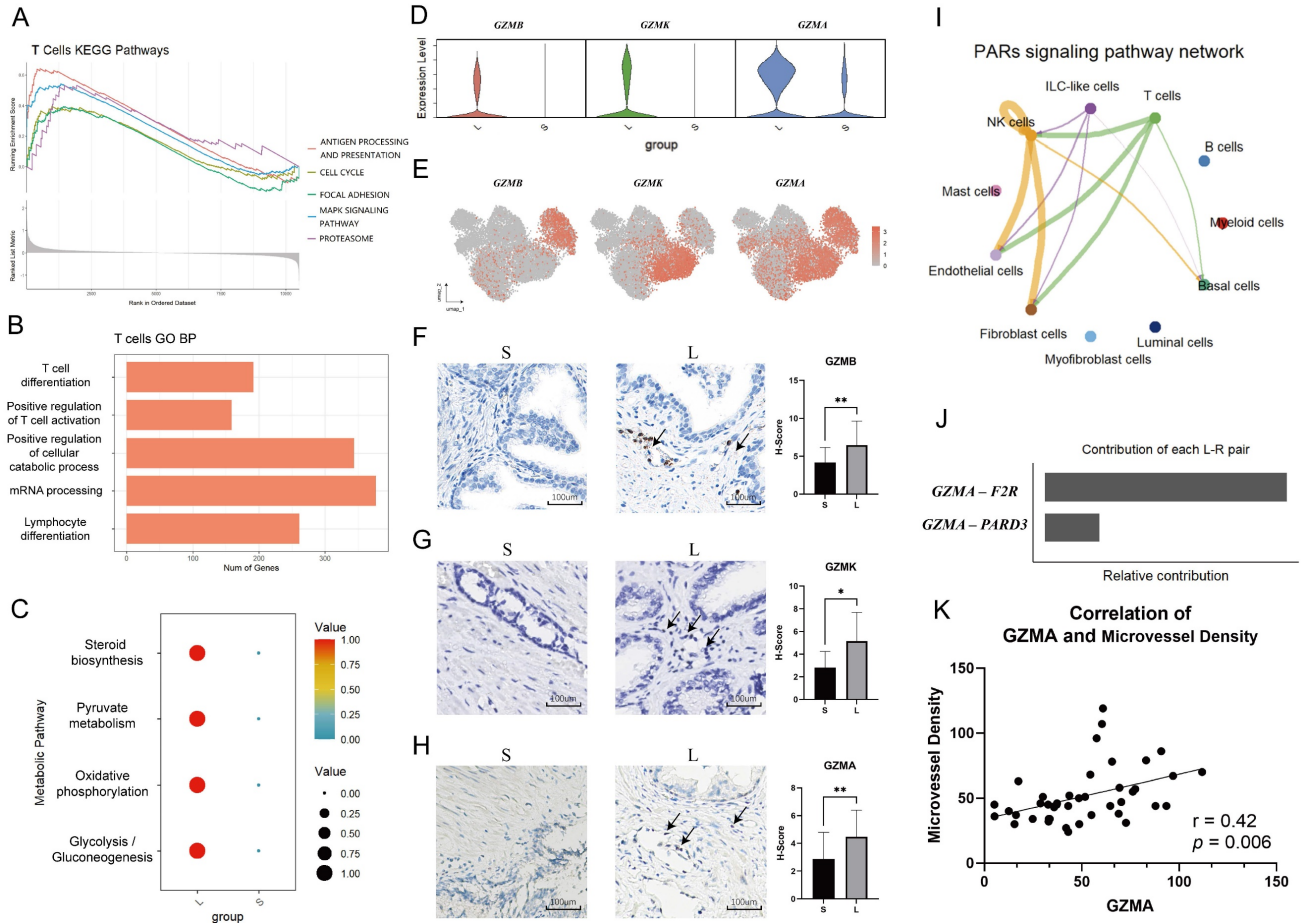
** Comparison of the functions of T cells between the large and small prostates. (A)** KEGG pathway plot of T cells between large and small prostates. The left side is large prostates, and the right side is the small prostates. **(B)** GO BP pathway plot of T cells in large and small prostates. A positive value of counts indicates that the relevant pathways are up- regulated in the large prostates. **(C)** Metabolic pathway plot of T cells in large and small prostates. **(D-E)** GZMB, GZMK and GZMA expression violin plot and gene plot of T cells in large and small prostates. **(F-H)** Representative pictures of IHC staining of GZMB, GZMK and GZMA in prostate transition zone tissue and statistical bar graphs, Right bars graph indicates statistical results of IHC. **(I)** Cell-to-cell communication of PARs signaling pathway in large prostates. **(J)** Specific ligand-receptor pairs contributing to the PARs signaling pathway in large prostates. **(K)** Correlation statistics between GZMA and microvessel density in prostates based on IHC. (* p<0.05; ** p≤0.01).

It is known that immune cells are recruited from peripheral blood to sites of inflammation 21. Furthermore, T cells exit quiescence to initiate clonal expansion, effector differentiation and enhanced metabolism when antigen stimulation occurs 22. We performed gene enrichment analyses of T cells to explore the functional differences between them in the large and small prostate. Kyoto Encyclopedia of Genes and Genomes (KEGG) enrichment analysis demonstrated that some immune-related and metabolic cell signaling pathways, including antigen processing and presentation, were upregulated in large prostates compared with small prostates (Fig. 4A). Gene Ontology (GO) enrichment analysis showed that T-cell differentiation, positive regulation of T-cell activation and other pathways were all upregulated to varying degrees in the large prostate (Fig. 4B). We further analyzed the metabolism of the large and small prostate, which revealed that metabolism was more vigorous in the large prostate (Fig. 4C). Therefore, based on the analysis of pathways related to antigen presentation, proliferation and metabolism, T cells in large prostates are more active than those in small prostates.

The significant function and weapon of the immune system is the ability of cytotoxic lymphocytes to kill virus-infected or transformed cells by apoptosis, which depends on the perforin-granzyme effector pathway 23. We found that there were more T cells secreting granzymes in the large prostate (Fig. 4D). Although CD8+ T cells secreting granzyme B (GZMB) and granzyme K (GZMK) were mostly concentrated in the large prostate, they were not in the same subclusters (Fig. 2A, 4E), as described in previous studies, which indicated that the effector cells in the large prostate still have different divisions of labor; some of the effector cells mainly have a killing function, while some are mainly secreting cytokines 23-24. Immunohistochemistry analysis demonstrated significant differences in the secretion of GZMB and GZMK between small and large prostates (Fig. 4F-H) (n = 20 in each group).

Furthermore, we found crosstalk between granzyme A (GZMA) and coagulation factor II thrombin receptor (F2R) on the surface of fibroblasts and endothelial cells in large prostates (Fig. 4I-K), suggesting that immune cells are involved in fibrosis and angiogenesis in the prostate 25-26. We also performed differential genetic analysis of NK cells in large and small prostates Fig. S4A). Fewer GZMA-secreting T and NK cells were also present in small prostates than in large prostates (Fig. 4D, 4H, S4B), but we did not find differences in the cytotoxicity of NK cells between large and small prostates (Fig. S4C).

### Anti-rejection drugs inhibit prostatic hyperplasia

**Figure 5:**
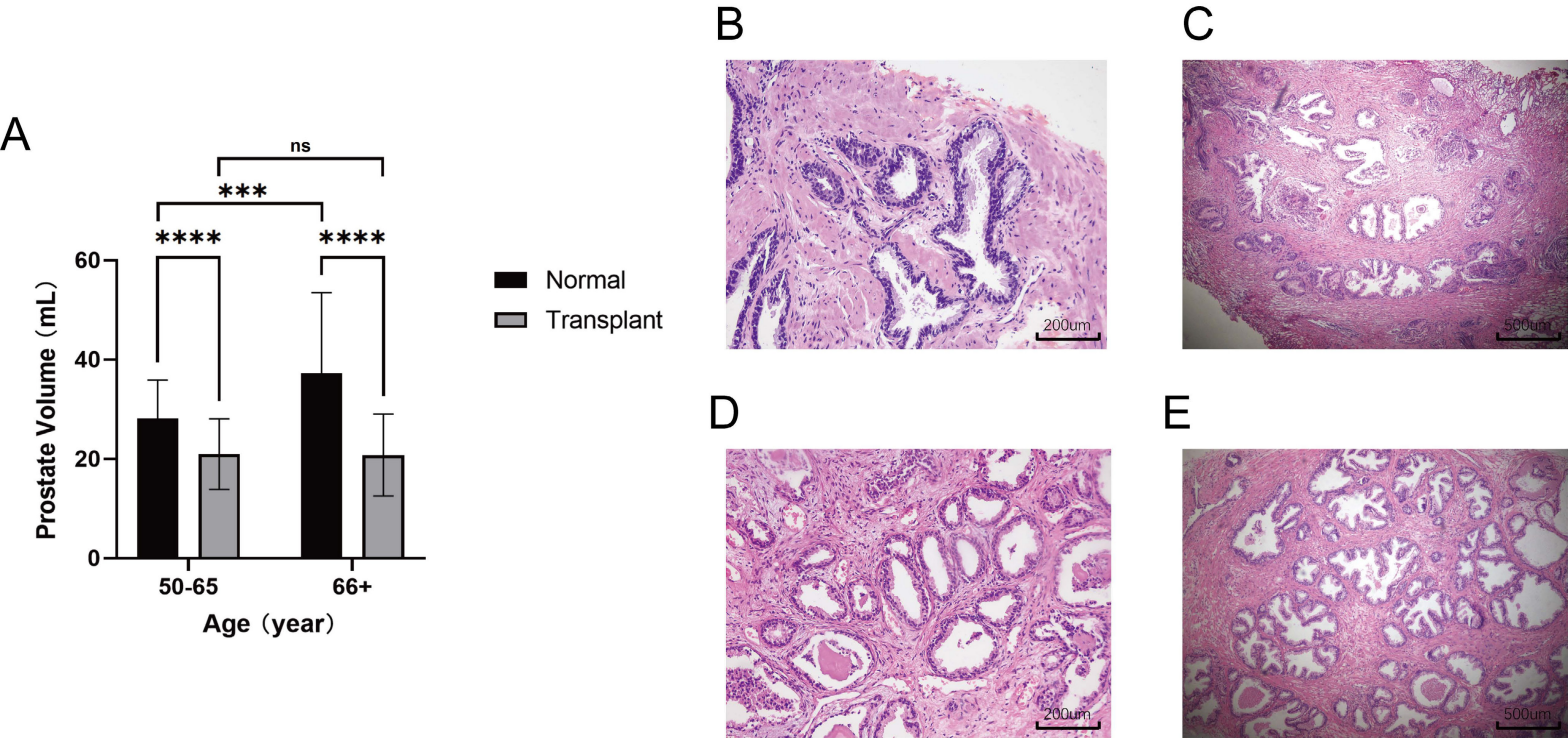
** Patients with anti-rejection drugs had smaller volumes of prostate. (A)** Comparison of prostate volume between renal transplant patients of different ages and the normal population. **(B-C)** HE-stained histopathological sections with different magnification of prostate transition zone in kidney transplant patients. **(D-E)** HE-stained histopathological sections with different magnification of prostate transition zone in normal prostate. (ns p≥0.05; *** p≤0.001; **** p≤0.0001).

Renal transplant patients require long-term administration of antixenobiotics, such as tacrolimus or rapamycin, which keep the immune system in a suppressed state and guarantee renal allograft survival (27-28. To validate the repression exerted on the prostate in the immunosuppressed state, an approved retrospective evaluation of patients from the Shanghai General Hospital was conducted to determine whether prostatic hyperplasia was inhibited in renal transplant patients who took antirejection drugs. Male patients with renal transplants over the age of 50 who underwent transabdominal B-mode ultrasound of the prostate from September 2021 to November 2022 were included (n = 67). Patients with a diagnosis of cancer were excluded from the study. A random sample of elderly males who underwent transabdominal B-mode ultrasound of the prostate in the medical examination center in November 2022 was used as a control (n = 100). Strikingly, the mean prostate volume in renal transplant patients was only 20.93 mL, which is far below 30 mL, the standard definition of a small prostate, and it was also significantly smaller than that in the normal group at different age levels. Consistently, the prostate volume did not change with age in renal transplant patients, whereas the prostate volume increased with age in the normal group. (Fig. 5A). The histological analysis of prostate transition zone tissue from two kidney transplant patients did not show any noteworthy alterations in comparison to the normal population (Fig. 5B-E). These findings provide robust evidence that immunosuppression impedes prostatic hyperplasia without causing considerable modifications in the cellular architecture of the prostate.

We conducted a thorough analysis of the medication regimen of the 60 kidney transplant patients enrolled in the study Table S2. The patients' medications primarily consisted of calcineurin inhibitors (CNIs) such as cyclosporine A (CsA) and tacrolimus (FK506), rapamycin, mycophenolate mofetil (MMF), corticosteroids, and other drugs. Although CNIs exhibit stronger immunosuppressive effects, rapamycin is less nephrotoxic and is associated with lower incidence rates of infection and malignancy in solid organ transplant patients 16, 28. Moreover, rapamycin preserves T-cell receptor (TCR) signaling pathway sensitivity in cells 28. Consequently, we chose rapamycin as the immunosuppressant to investigate the relationship between immunosuppression and BPH.

### Rapamycin or low-dose IL-2 inhibits BPH in mice

**Figure 6:**
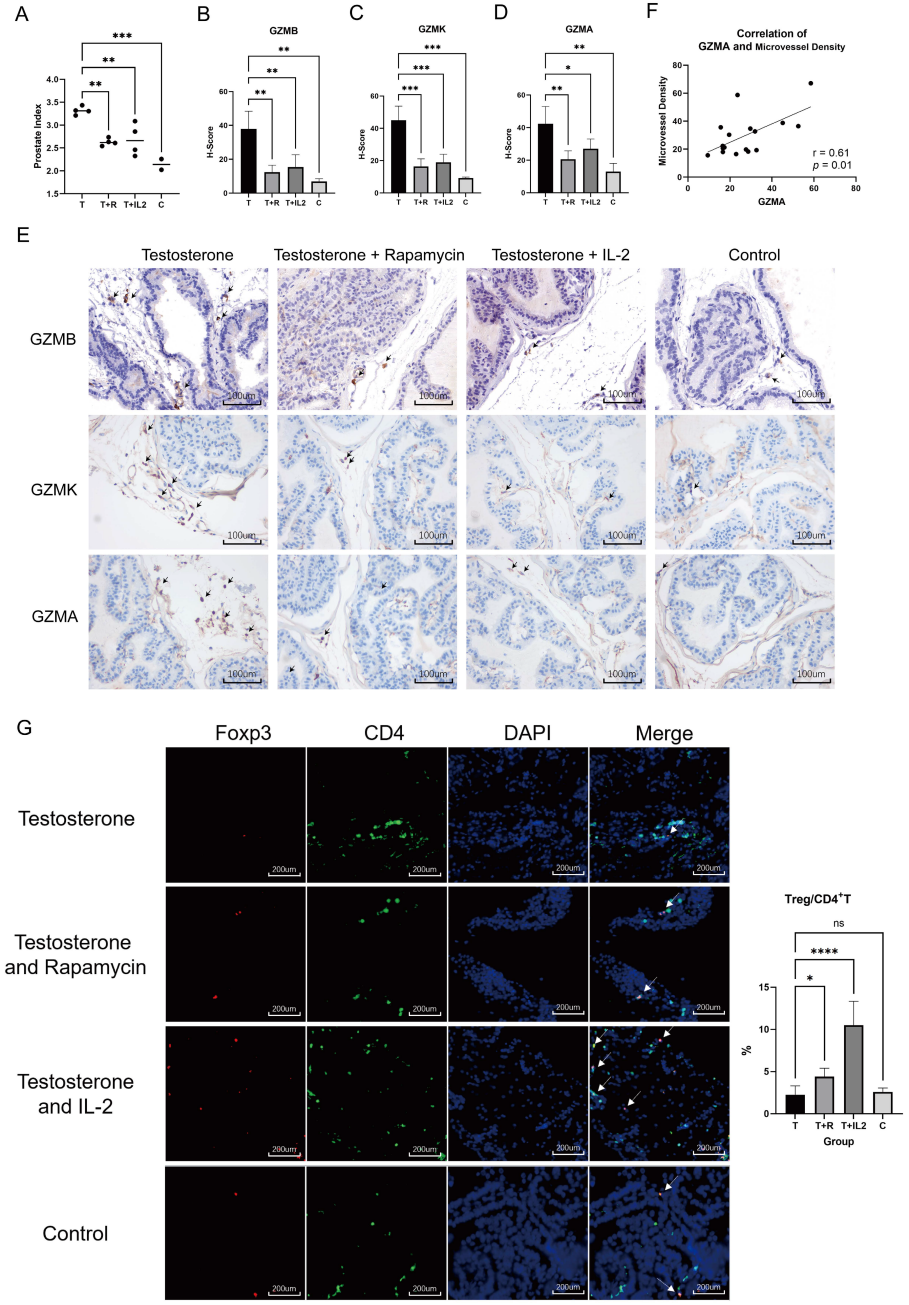
** Testosterone propionate induced BPH was suppressed by rapamycin or low- dose IL-2 in mice. (A)** Comparison of the prostate index of mice in the BPH module only injected with testosterone propionate, the rapamycin group infused simultaneously with modeling, and the low-dose IL-2 group injected simultaneously with modeling, as well as the normal group. T: Testosterone propionate; R: Rapamycin; C: Control. **(B-D)** Statistical bar graphs of IHC staining of GZMB, GZMK and GZMA in mice prostate. **(E)** Representative pictures of IHC staining of GZMB, GZMK and GZMA in mice prostate. **(F)** Correlation statistics between GZMA and microvessel density in mouse prostates based on IHC. **(G)** Immunofluorescence of mice prostate displays different enrichment of Tregs, and right statistical bar graph indicates statistical results of immunofluorescence. (* p<0.05; ** p≤0.01; *** p≤0.001; **** p≤0.0001).

Rapamycin, a commonly used immunosuppressant, has lower infection and tumor incidence in the clinic and has been widely used to prevent rejection after organ transplantation 27-29. Subcutaneous injection of testosterone propionate into adult male mice has been shown to induce the occurrence of BPH 30. To evaluate the effects of immunosuppressive status on BPH, we used rapamycin to intervene in mice that developed BPH. Adult C57B6/L male mice were subjected to castration followed by daily subcutaneous injection of testosterone propionate at 5 mg/kg with or without daily gavage of rapamycin at 1 mg/kg for one month (n = 4 in each group). Normal non-treated mice were used as controls (n = 2 in the control group). The results of prostate index comparison in mice showed that rapamycin alleviated prostate hyperplasia in mice (Fig. 6A). Histological evaluation of prostate tissue demonstrated that rapamycin improved the degree of glandular cell hyperplasia and decreased granzyme expression in the tissue (Fig. 6B-E). While GZMA was reduced, we also noticed a decrease in microvessel density in the mouse prostate (Fig. 6F). This indicated that the immunosuppressive environment mediated by rapamycin inhibits testosterone-induced BPH *in vivo*.

Low-dose IL-2 can directionally induce the augmentation of Tregs and has been applied in clinical trials for the treatment of AID such as systemic lupus erythematosus (SLE). Compared with conventional immunosuppressive agents, low-dose IL-2 possesses several advantages, such as fewer side effects and better patient tolerance 14, 31. C57B6/L mice were subjected to castration followed by daily subcutaneous injection of testosterone propionate at 5 mg/kg and daily intraperitoneal injection of IL-2 at 25 μg/kg (IL-2 was given on days 1-5, 9, 13, 17, 21, 25, 29, 30) for one month (n = 4 in the IL-2 group). It was experimentally confirmed that low-dose IL-2 repressed testosterone-induced BPH *in vivo* while decreasing granzyme expression in the prostate (Fig. 6B-E). Immunofluorescence further confirmed that mice treated with rapamycin and low-dose IL- 2 had a greater CD4+Foxp3+T/CD4+T ratio than the testosterone propionate module group in the prostate (Fig. 6G). Previous studies have established that the administration of rapamycin results in an elevated count of circulating Tregs in the peripheral blood of patients with rheumatoid arthritis or transplant recipients 32-33. Based on this evidence, we think that rapamycin treatment similarly induces an augmentation of Treg cell populations within the prostate of mice. These results confirmed that an immunosuppressive environment can suppress prostate hyperplasia in the presence of androgens, whereas Tregs are able to induce this immunosuppressive microenvironment.

## Discussion

Previous studies have primarily compared old prostate tissue to young prostate tissue, explored differences between the transitional zone and peripheral zone of prostate tissue, or revealed the difference between hyperplastic tissue and normal tissue surrounding prostate cancer 11, 34-35. In this study, we conducted comprehensive sequencing of all the cell types found in prostate tissue from older men without prostate cancer or BPH medication, and we compared tissue from large and small prostates. This approach provides a more representative comparison of normal prostate tissue than previous studies, which used prostate cancer tissue for comparison. This study is of considerable importance for comprehending the mechanisms of BPH in addition to age and sex hormones.

Our findings indicate differences in the cellular composition of large and small prostates. Previous research has suggested that BPH is characterized by an increase in stromal and epithelial cell proliferation 36-37, and our data show that large prostates have a higher number of fibroblasts, myofibroblasts, and vascular endothelial cells, with a lower number of luminal and basal cells. BPH is known to involve the fundamental remodeling of cell types 36, and our results suggest that large prostate tissue may possess more stroma, which could be a contributing factor to the development of BPH. Our research also highlights that the prostate is an immunocompetent organ with a complex immune system 38, as evidenced by the high number of immune cells in the tissue. Moreover, we found that the immune cell composition between small and large prostates differs significantly in terms of function, which creates a distinct immune microenvironment that may play a vital role in the pathogenesis of BPH.

The prostate contains naive T cells in both large and small prostates; however, their differentiation pathways are distinct. The small prostate has a high number of infiltrating immune cells, but most of these cells lack the expression of granzyme, indicating that they do not exhibit direct killing function. On the other hand, in the large prostate, there is upregulation of activation pathways of T cells, with a more metabolically active state and higher expression of granzymes. Following the recruitment of immune cells to the prostate tissue, these cells undergo various biological processes, including activation and differentiation, ultimately leading to the immune response of target cell killing mediated by cytotoxic lymphocytes via granzymes. Granzyme levels serve as a direct indicator of the immune activity mediated by these cells 23, 39. While previous studies have focused on the correlation between BPH and cytokines in the immune system 6, our research has shed light on the association between granzymes and BPH.

GZMB and GZMA serve as critical effectors in inducing target cell death, with GZMB being the more potent apoptosis-promoting granzyme. Even at nanomolar concentrations, GZMB can rapidly induce target cell apoptosis, while the effects of GZMA are slower. Furthermore, GZMB has a profibrotic impact and has been linked to several fibrotic diseases, potentially accounting for the extensive stromal proliferation observed in large prostates 40-41. In addition, our investigation revealed that in the prostate, GZMA stimulates F2R receptors on vascular endothelial cells, leading to blood vessel growth and further exacerbating the progression of BPH 25-26. Prior research has indicated a higher occurrence of senescent cells within BPH tissues 42. While GZMK, functioning as a granzyme, does not possess the ability to directly induce target cell death, it can facilitate cellular senescence 24. This, in turn, can augment immune cell infiltration and escalate the local immune response, ultimately leading to the establishment of an activated immune microenvironment. The elevated number of granzymes in large prostates is a direct reflection of the presence of more cytotoxic lymphocytes and a more activated immune microenvironment. These recruited immune cells can produce TGF-β and interleukins, creating a persistent inflammatory immune microenvironment and promoting the progression of BPH 37.

Kidney transplant recipients who are prescribed immunosuppressive drugs do not exhibit significant prostate enlargement. Unlike the general population, kidney transplant patients need to take multiple immunosuppressive drugs, including CNIs, rapamycin, MMF, glucocorticoids, and others, for prolonged periods to ensure the survival of the transplanted kidney 43. Such drugs strongly suppress the function of the patient's systemic immune system while having few adverse effects on the patient's health. Consequently, the immune microenvironment of the prostate in kidney transplant patients is relatively suppressed. This finding suggests that under conditions of immunosuppression, there is an absence of prostate hyperplasia, the size of the prostate does not increase with the increased age of the patient, and there is no significant alteration in the prostate cellular makeup in comparison to the general population. Although previous studies have extensively demonstrated that inflammation may be one of the causes of BPH, our clinical data provide direct evidence that immunosuppression can impede the progression of BPH and that an activated immune microenvironment in the prostate is necessary for prostate volume growth 6.

Treg cells are a type of immune cell that are known for their suppressive function and have been extensively studied for their role in regulating the immune microenvironment in various tissues 13, 15. Previous research has suggested that patients with BPH exhibit a reduced proportion of Treg cells in their peripheral blood when compared to healthy young individuals 20. However, in our study, we observed no significant difference in the proportion of Treg cells in the peripheral blood of elderly patients with varying prostate volumes. These findings suggest that there may be a mechanism of Treg recruitment or proliferation in small volume prostates. Inactive Treg cells present in the peripheral blood may be continuously recruited to the small prostate, where they are stimulated to activate and proliferate, ultimately contributing to the establishment of an immunosuppressive microenvironment.

For the first time, we present evidence that BPH can be effectively suppressed through *in vivo* immunosuppressive therapy. Currently, the pathogenesis of BPH is primarily attributed to inflammation and hormonal factors 6, 11, 30. We have demonstrated in animal experiments that the injection of high doses of testosterone induces the formation of an activated immune microenvironment in the mouse prostate and promotes the development of BPH. Our findings suggest that the administration of rapamycin decreases the content of granzyme and promotes an increase in the proportion of Tregs in the prostate, which implies the suppression of the secondary immune activation state. Our results show that the progression of BPH can be arrested through immune system suppression, even when mice still have extremely high concentrations of serum testosterone. These findings further support the association between the activated immune microenvironment of the prostate and the development of BPH. While the role of androgen and its receptors in BPH is not yet fully understood, we hypothesize that the persistently activated immune state in the prostate may be one of the mechanisms underlying the failure of BPH to resolve in some patients despite taking 5α-Reductase Inhibitors (5ARIs) 30.

Upon administering rapamycin to mice, we observed an increase in Tregs in the prostate, and low-dose IL-2 stimulation of Treg proliferation resulted in a suppressive immune microenvironment in the prostate. Our experiments showed that Treg proliferation in the prostate induces a suppressive immune microenvironment, reduces granzyme expression in prostate tissue, and prevents benign prostatic hyperplasia progression in mice. Rapamycin has an advantage over CNIs due to its lower incidence of infection and malignancy, which makes it better tolerated by patients 28. The possibility of intermittent administration of rapamycin is currently being explored to minimize immunosuppressant toxicity while suppressing immune function and limiting metabolism. Rapamycin and low- dose IL-2 have been experimentally used to treat AID with positive therapeutic effects 14, 42. Consequently, we believe that rapamycin and low-dose IL-2 have broader clinical application potential than CNIs for establishing a prostate immune microenvironment that inhibits the progression of BPH.

Several trials evaluating the use of nonsteroidal anti-inflammatory drugs (NSAIDs) to alleviate symptoms associated with BPH have demonstrated some favorable outcomes; nevertheless, sustained benefits appear to be limited 45-46. The anti-inflammatory effects of NSAIDs are attributed to their inhibition of prostaglandin synthesis and white blood cell aggregation. Nevertheless, unlike rapamycin and low-dose IL-2, which can fully reverse the activated immune microenvironment in the prostate, NSAIDs lack this capability. Therefore, it is essential to further explore the potential of immune-modulating drugs such as rapamycin and low-dose IL-2 in managing BPH patients who do not respond to 5ARIs. Alternatively, a combination therapy of immunosuppressants with 5ARIs and α- receptor blockers may provide a more effective therapeutic strategy and result in better clinical outcomes.

A total of 6 prostate samples were subjected to sequencing, which was subsequently validated through histologic examination. Despite the inclusion of more samples for histologic validation, the smaller number of sequenced samples could be subject to potential biases and limit further exploration of Treg subpopulations. The retrospective nature of peripheral blood data of patients with BPH and prostate data of kidney transplant patients poses several limitations. Moreover, renal transplant patients receiving long-term rapamycin or other immunosuppressive agents are also taking glucocorticoids, and their effects on BPH are unknown. In addition, a genuine murine model for BPH has yet to be developed, and the role of immunosuppression in inhibiting BPH progression *in vivo* remains to be confirmed. Last, despite our demonstration of Tregs' ability to suppress BPH within the immunosuppressive environment, the relationship between the immunosuppressive environment and BPH and the mechanism underlying the existence of a relatively immunosuppressive environment in the prostate of the general population are not fully understood.

Our findings demonstrate the crucial role of the immune microenvironment in prostate volume regulation. Specifically, the persistent activation state of the prostate immune microenvironment is a prerequisite for the continuous enlargement of prostate volume. Conversely, inhibiting the immune activation state of the prostate can prevent prostate volume growth. We further observed that differences in Treg proportions in the prostate are a key factor in creating an immunosuppressive microenvironment in the normal population. The results of our animal experiments highlight the effectiveness of rapamycin and low- dose IL-2 in halting the progression of BPH, and future research will explore their clinical applications for BPH patients.

## Conclusions

An activated immune microenvironment is necessary for prostate volume growth and that Tregs can reverse this immune activation state, thereby inhibiting the progression of BPH. Rapamycin and low-dose IL-2 in halting the progression of BPH and holding potential for future clinical treatment of BPH.

## Supplementary Material

Supplementary figures and tables.
