# Supplementary material for: Rapamycin and Low-dose IL-2 Mediate an Immunosuppressive Microenvironment to Inhibit Benign Prostatic Hyperplasia
Source: Int J Biol Sci. 2023 Jul 3;19(11):3441–55. doi: 10.7150/ijbs.85089 (PMC10367549; doi:10.7150/ijbs.85089)
Supplement: Supplementary file 1 — Supplementary figures and tables. [file ijbsv19p3441s1.pdf]

Supplemental material

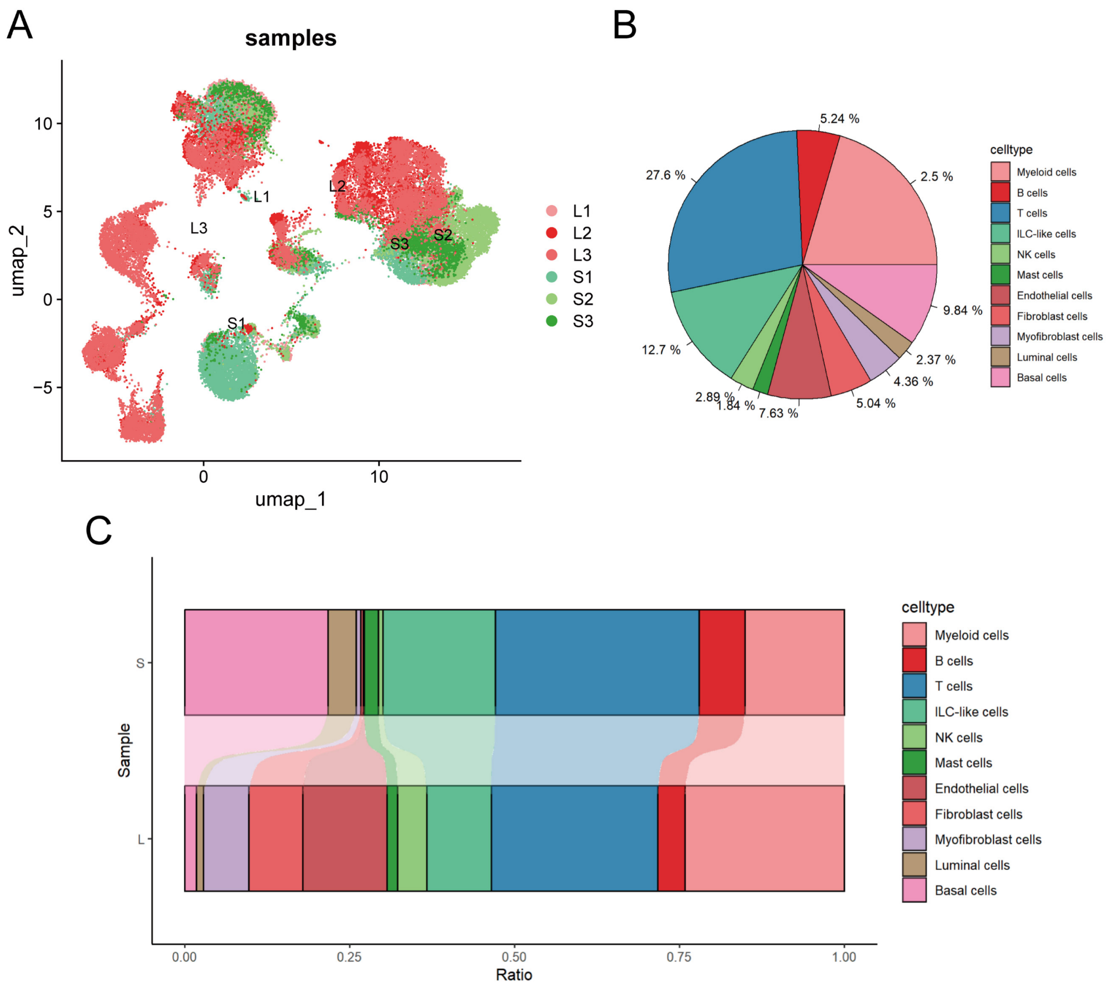

Figure S1. (A) All cells of prostates are divided according to their prostate size original idents. **(B)** Pie chart of the all prostate samples' cell ratio. **(C)** Bar graph of large prostate samples, and small prostate samples' cell ratio.

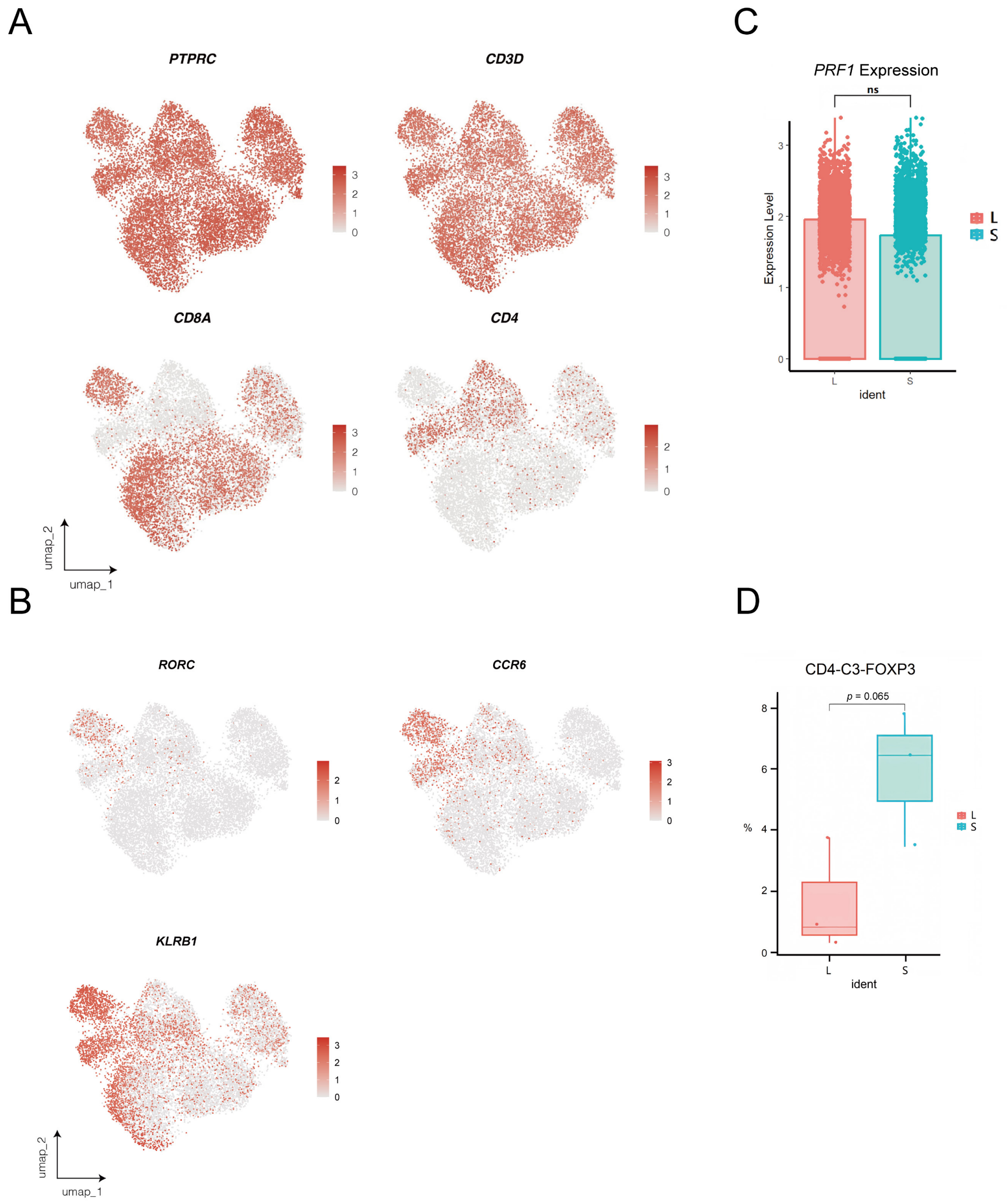

Figure S2. (A) Specific gene expression plot of T cells. **(B)** RORC, CCR6 and KLRB1 expression plot of T cells. **(C)** Single-cell data revealed that there was no difference in PRF1 expression between large and small prostate T cells. (ns p≥0.05) **(D)** Single-cell data suggest small prostates contain more Tregs (p=0.065).

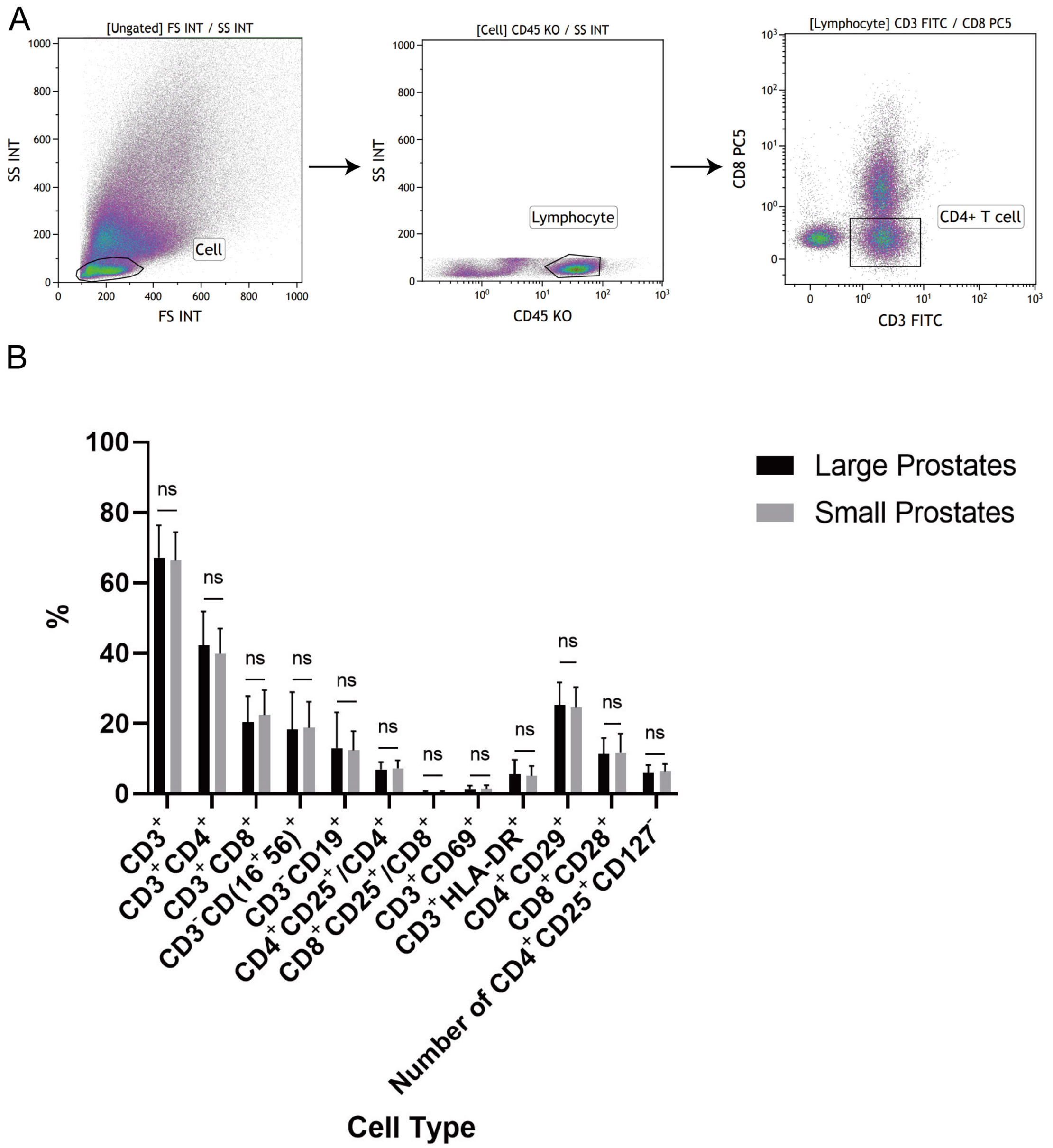

Figure S3. (A) Detailed standard flow cytometry strategy for detection of Tregs in the prostate. **(B)** Comparison of flow cytometry results of peripheral blood immune cells in patients with large and small prostates. (ns p≥0.05)

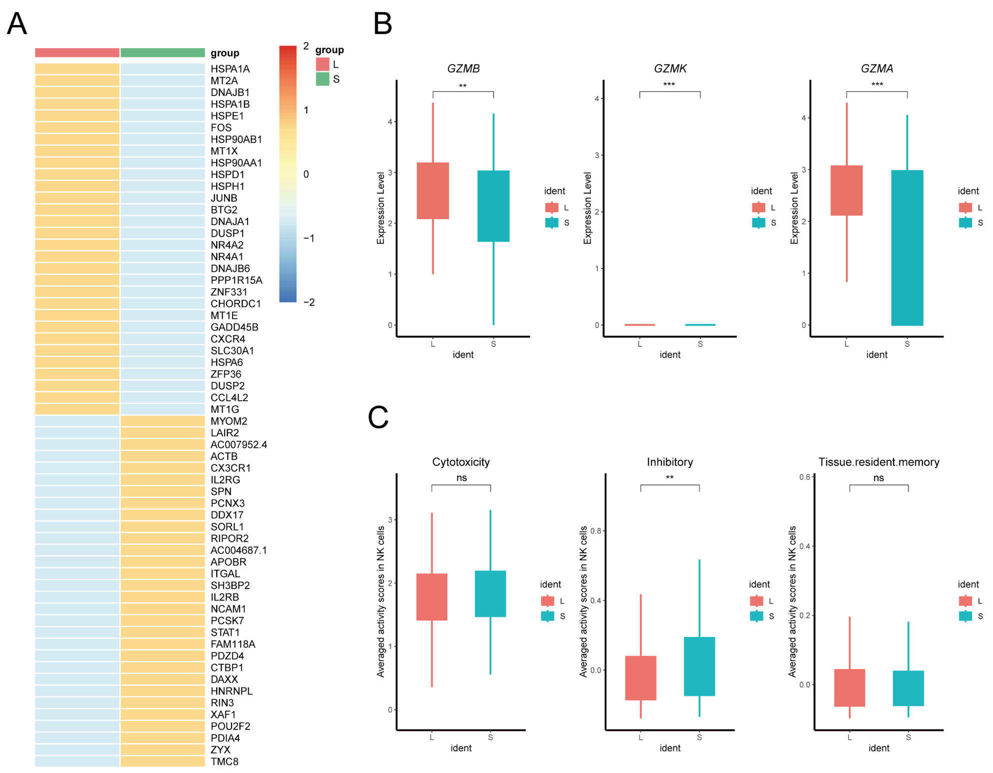

Figure S4. (A) Differential genes in NK cells in large and small prostates. **(B)** GZMB, GZMK and GZMA expression box plot of NK cells in large and small prostates. **(C)** Averaged activity scores for the indicated gene signatures of NK T cells in large and small prostates. (ns p≥0.05; ** p≤0.01; *** p≤0.001)

Table S1. Peripheral Blood Immune Cell Flow Cytometry Detection of patients.

Patient	Peripheral Blood Immune Cell Flow Cytometry Detection											
	CD3+	CD3+CD4+CD8-	CD3+CD4-CD8+	CD3-CD(16+56)+	CD3-CD19+	CD4+CD25+/CD4+	CD8+CD25+/CD8+	CD3+CD69+	CD3+HLA-DR+	CD4+CD29+	CD8+CD28+	Number of CD4+CD25+CD127-
Small Prostate 1	79.12	37.85	36.07	5.87	11.73	4.5	0.01	1.92	4.55	23.29	25.49	3.3
Small Prostate 2	55.77	34.99	19.49	29.59	11.24	8.78	0.25	1.12	3.21	25.12	8.01	8.18
Small Prostate 3	65.75	37.8	23.55	17.45	14.95	6.22	0.64	3.8	7.65	24.5	11.2	6
Small Prostate 4	N/A	N/A	N/A	N/A	N/A	N/A	N/A	N/A	N/A	N/A	N/A	N/A
Small Prostate 5	79.67	47.36	17.15	5.97	10.17	1.99	0.01	2.91	2.96	28.68	7.96	1.09
Small Prostate 6	71.15	34.45	31	22.8	5.23	6.68	0.32	5.32	8.76	27.19	9.92	5.59
Small Prostate 7	67.25	37.76	26.65	17.91	13.13	7.73	0.72	1.46	10.93	26.28	21.19	7.46
Small Prostate 8	N/A	N/A	N/A	N/A	N/A	N/A	N/A	N/A	N/A	N/A	N/A	N/A
Small Prostate 9	74.7	35.92	35.25	17.5	7.35	6.73	0.41	2	4.8	22.15	15.5	6.6
Small Prostate 10	61	45.06	13.43	18.41	16	5.5	0.33	1.47	11.75	33.07	10.11	5.26
Small Prostate 11	55.48	32.57	18.6	15.76	24.76	8	0.26	0.6	2.45	28.59	11.12	7.92
Small Prostate 12	75.61	47.55	25.82	12.24	8.26	2	0.01	1.73	7.11	18.83	12.89	0.7
Small Prostate 13	N/A	N/A	N/A	N/A	N/A	N/A	N/A	N/A	N/A	N/A	N/A	N/A
Small Prostate 14	N/A	N/A	N/A	N/A	N/A	N/A	N/A	N/A	N/A	N/A	N/A	N/A
Small Prostate 15	N/A	N/A	N/A	N/A	N/A	N/A	N/A	N/A	N/A	N/A	N/A	N/A
Small Prostate 16	N/A	N/A	N/A	N/A	N/A	N/A	N/A	N/A	N/A	N/A	N/A	N/A
Small Prostate 17	65.65	39.09	21.09	17.5	14.4	8.89	0.23	0.6	9.7	27.11	8.35	5.22
Small Prostate 18	N/A	N/A	N/A	N/A	N/A	N/A	N/A	N/A	N/A	N/A	N/A	N/A
Small Prostate 19	76.8	36.45	31	8.7	11.45	7	0	1.2	9.15	24.5	13.65	5.72
Small Prostate 20	76.7	46.29	24.77	8.52	10.04	2.64	0.01	1.89	5.82	25.18	7.52	0.36
Small Prostate 21	66.2	42.05	17	15.25	16.65	7.25	0.59	1.75	7.5	32.85	10.6	6.51
Small Prostate 22	69.14	33.25	20.05	21.01	8.71	7.02	0.25	1.4	1.61	19.28	11.16	6.66
Small Prostate 23	80.86	49.15	24.5	7.73	9.05	6.55	0.6	0.82	4.45	34	10.03	5.91
Small Prostate 24	59.05	38.5	21.8	29.35	9.35	9.09	1.15	0.8	6.05	28.45	5.2	6.43
Small Prostate 25	48.91	29.62	14.69	23.55	24.82	10.47	0.33	1.56	7.3	20.46	8.76	8.96
Small Prostate 26	58.02	33.46	22.89	25.99	13.23	3.93	0.01	0.57	1.57	23.4	6.53	2.94
Small Prostate 27	59.7	47.1	10.7	18.25	19.05	10	0.47	1.1	2.05	27.89	8.13	9.88
Small Prostate 28	71.3	42	28.3	19	8.5	7.71	0.18	0.6	5.55	23.95	14.35	7.17
Small Prostate 29	74.85	48.69	20.9	10.65	12.75	6.65	0.74	0.95	2.55	23.55	8.75	4.36
Small Prostate 30	68.25	51.15	12.8	17.1	12.55	8.6	0.39	0.7	3.8	27.05	9.3	6.98
Small Prostate 31	67.7	50.61	14.98	18.9	11.55	6.87	0.23	0.7	1.55	26.26	9.77	6.4
Small Prostate 32	63.9	29.8	34	15.85	17.8	6.71	0.29	0.75	6.2	17.45	21.85	6.61
Small Prostate 33	59.45	30.3	22.8	34.8	5.15	8.91	0.1	1.5	7.2	15.9	9.4	6.31
Small Prostate 34	48.75	40.5	7.9	40.15	9.35	7	0.1	0.95	1.6	26.45	4.4	6.6
Small Prostate 35	N/A	N/A	N/A	N/A	N/A	N/A	N/A	N/A	N/A	N/A	N/A	N/A
Small Prostate 36	67.64	31.88	34.63	23.17	6.47	10	0.14	0.47	7.46	21.03	10.42	9.76
Small Prostate 37	59.7	35.1	23.9	28.6	10.05	8.83	0.42	0.9	4.5	23.75	12.45	8.52
Small Prostate 38	58.4	46.3	11.9	17.6	20.85	7.99	0.84	0.55	1.45	23.65	8.15	7.35
Small Prostate 39	64.95	28.65	32.85	23	8.7	12.74	0.46	1.15	6.15	22.35	30.35	8.57
Small Prostate 40	70.45	54.05	15.61	21.04	7.48	9.37	0.93	0.64	4.55	37.18	8.44	8.9
Small Prostate 41	74.7	39.95	30	15.9	8.3	8.51	0.5	2.1	8.5	22.8	16.95	6.14
Small Prostate 42	60.87	42.34	18.5	25.63	10.38	2.3	0.01	2	2.88	19.9	8.1	1.6
Small Prostate 43	75.71	45.51	21.93	8.27	15.09	7.5	0.85	0.5	2.6	26	19.75	7.03
Small Prostate 44	73.25	34.48	26.52	17.5	8.2	7.2	0.1	1.2	9.15	24.55	15.31	7.02
Small Prostate 45	N/A	N/A	N/A	N/A	N/A	N/A	N/A	N/A	N/A	N/A	N/A	N/A
Small Prostate 46	68.73	40.4	26.59	20.55	8.06	4	0.61	2.75	6.6	29.96	11.07	3
Small Prostate 47	N/A	N/A	N/A	N/A	N/A	N/A	N/A	N/A	N/A	N/A	N/A	N/A
Small Prostate 48	58.4	35.47	20.58	14.61	24.02	9.34	0.7	0.48	1.96	31.11	14.03	8.89
Small Prostate 49	68.3	35.5	31.1	20	8.85	9.2	0.16	0.8	3.2	22.54	13.58	8.97
Small Prostate 50	59.8	31.25	26.55	29.95	6.4	10.08	0	3.1	6.8	18.85	9.65	9.52
Small Prostate 51	64.15	30.25	31.3	23.55	10.9	5.5	0.64	3.25	4.5	14.6	9.05	5.09
Small Prostate 52	75.35	45	27.7	15.95	6.45	6.67	0.36	1.8	3.35	32.95	10.9	5.82
Small Prostate 53	69.64	44.61	19.51	19.54	7.64	8.2	0.78	1.13	1.42	17.29	8.05	7.53
Small Prostate 54	N/A	N/A	N/A	N/A	N/A	N/A	N/A	N/A	N/A	N/A	N/A	N/A
Small Prostate 55	59.85	37.12	21.23	27.75	10.4	9.8	0.44	1.7	3.55	23.31	10.32	7.05
Small Prostate 56	62.73	45.83	12.41	11.54	23.55	7.5	0.81	0.78	1.55	24.69	9.85	7.11
Small Prostate 57	78.23	44.45	26.55	13.26	6.5	6.6	0.56	1.46	1.86	23.2	23.3	3.16
Small Prostate 58	59.5	43.45	11.45	28.1	9.3	7.5	0.1	3.8	3.9	32.2	10.55	7.19
Small Prostate 59	N/A	N/A	N/A	N/A	N/A	N/A	N/A	N/A	N/A	N/A	N/A	N/A
Small Prostate 60	N/A	N/A	N/A	N/A	N/A	N/A	N/A	N/A	N/A	N/A	N/A	N/A
Small Prostate 61	N/A	N/A	N/A	N/A	N/A	N/A	N/A	N/A	N/A	N/A	N/A	N/A
Small Prostate 62	68.84	49.25	15.15	13.26	13.65	7.92	2.31	1.17	7.39	32.42	14.15	7.4
Small Prostate 63	N/A	N/A	N/A	N/A	N/A	N/A	N/A	N/A	N/A	N/A	N/A	N/A
Small Prostate 64	N/A	N/A	N/A	N/A	N/A	N/A	N/A	N/A	N/A	N/A	N/A	N/A
Small Prostate 65	65.03	29.11	29.29	7.14	24.7	6.5	0.16	0.61	7.47	17.78	14.85	6.11
Small Prostate 66	52.11	27.42	19.06	22.62	21.48	10	0.1	0.17	1.13	3.45	2.35	9.29
Small Prostate 67	N/A	N/A	N/A	N/A	N/A	N/A	N/A	N/A	N/A	N/A	N/A	N/A
Small Prostate 68	60.25	41.75	16.3	19.45	17.4	4.91	0	0.7	10.5	29.05	10.95	4.6
Small Prostate 69	75.75	55.38	19.02	11.44	12.22	7.18	1	1.35	5	22.15	10.9	6.54
Small Prostate 70	N/A	N/A	N/A	N/A	N/A	N/A	N/A	N/A	N/A	N/A	N/A	N/A
Large Prostate 1	N/A	N/A	N/A	N/A	N/A	N/A	N/A	N/A	N/A	N/A	N/A	N/A
Large Prostate 2	72.25	41.1	22.7	12.05	13.25	6.72	0	1.55	1.5	20.8	17.15	6.34
Large Prostate 3	73.32	35.02	35.32	22.14	3.64	7.54	0.46	2.27	5.45	26.15	16.75	3.33
Large Prostate 4	69.5	43.8	20.95	15.4	13.3	7.5	0.24	1.75	2.9	23.05	13.25	7.46
Large Prostate 5	70	44.75	24.05	19.8	8.2	7	0.1	0.8	12.15	24.2	7.85	6.8
Large Prostate 6	71.05	48	19.85	15.85	10.2	8.65	0.5	0.4	3	30.45	17.05	5.08
Large Prostate 7	N/A	N/A	N/A	N/A	N/A	N/A	N/A	N/A	N/A	N/A	N/A	N/A
Large Prostate 8	65.4	43.8	16.3	22.9	8	7.42	0.31	1.1	12.35	22.75	11.1	4.5
Large Prostate 9	66.55	37.4	21.85	11.7	19.6	7.5	0.23	3.7	4.65	25	10.35	7.3
Large Prostate 10	66.63	48.3	13.25	17.45	12.15	7.14	1.13	1.05	6.15	35.25	8.45	6.97
Large Prostate 11	71.5	44.75	23.65	14.3	10	7.92	0.3	1.35	5.75	22.8	7.75	7
Large Prostate 12	64.85	50.69	13	20.45	11.35	7.48	0	0.55	6.8	23.85	9.5	7.02
Large Prostate 13	76.2	46.35	27.9	14.7	6	8	0.54	1	0.9	19.59	15.17	7.98
Large Prostate 14	79.1	46.9	12.15	14.15	5.65	6.29	0.41	1.15	1.35	15.15	4.95	5.27
Large Prostate 15	70.91	51.41	18.97	9.67	16.27	3.4	0.27	1.73	5.32	23.88	11.27	2
Large Prostate 16	60.49	39.95	19.05	23.01	14.99	4.51	0.52	0	0.71	27.76	13.71	4.45
Large Prostate 17	52.73	40.24	8.54	29.94	16.84	6.74	0.1	0.9	3.2	18.15	5.95	4.2
Large Prostate 18	N/A	N/A	N/A	N/A	N/A	N/A	N/A	N/A	N/A	N/A	N/A	N/A
Large Prostate 19	76.42	59.34	11.31	9.49	9.15	6.7	0.42	0.81	2.91	29.38	5.98	4.87
Large Prostate 20	77.05	57.52	14.11	12.85	8.85	6	0.34	1	7.45	26.02	7.11	5.77
Large Prostate 21	65.24	41.25	19.35	19.13	12.72	6.2	0.26	1.28	3.5	16.81	6.76	6.18
Large Prostate 22	66.55	44.9	13.3	21.05	10.5	9	0.38	1	2.35	20.15	9.4	8.7
Large Prostate 23	69.85	27	34.1	18.4	7.05	9	0.44	3.35	11.6	20.54	16.75	8.91
Large Prostate 24	73.5	36.35	31.1	12.8	10.85	9.08	0.16	1.4	2.45	27.44	25.49	8.56
Large Prostate 25	79.95	58.5	20.05	12.2	5.35	7	0.25	0.8	2.15	40.05	13	6.34
Large Prostate 26	64.12	33.5	13.9	9.98	22.62	2.8	0.01	0.68	1.26	24.5	9.95	2
Large Prostate 27	71.59	41.01	24.12	6.26	20.39	1.96	0.1	2.2	3.58	25.79	19.79	1.08
Large Prostate 28	77.16	64.29	10.29	10.17	10.07	7.2	0.1	2.64	6.26	42.24	3.06	5.4
Large Prostate 29	56.75	20.65	27.3	35.2	6.9	9.69	0.18	0.6	6.95	16.9	8.1	6.74
Large Prostate 30	69.87	32.18	35.6	19.09	8.11	11	0.1	1.81	9.91	19.05	9.25	10.41
Large Prostate 31	75.69	50.87	19.8	12.79	8.51	2.54	0.25	2.43	5.93	25.67	9.52	1.61
Large Prostate 32	50.1	17.09	32.21	41.05	4.25	5.02	0.03	3	4.6	13.37	13.04	4.68
Large Prostate 33	75.2	38.3	28.35	10.9	12.2	6.83	1.23	2.7	7.9	28.38	17.65	6.67
Large Prostate 34	63.05	36.1	24.5	21.5	13.25	6	0.41	0.95	3.2	27.22	15.64	5.43
Large Prostate 35	75.7	32.4	43.7	11.65	10.55	8.64	0.55	0.35	3.25	21	21.8	7.95
Large Prostate 36	63.35	49.1	11.9	29.15	5	8.59	0.84	0.75	3.2	24.68	10.18	8.41
Large Prostate 37	64.14	38.21	21.28	24.55	8.89	7.24	0.22	2.51	4.54	32.72	10.09	6.51
Large Prostate 38	N/A	N/A	N/A	N/A	N/A	N/A	N/A	N/A	N/A	N/A	N/A	N/A
Large Prostate 39	76.2	46.6	27.5	7.8	13.5	7.08	0	0.15	4.1	31.05	10.95	5.6
Large Prostate 40	70.7	38	27.25	18.4	10.05	6.66	0.18	1.65	12.2	32.5	14.25	6.6
Large Prostate 41	42.22	35.65	6	52.32	2.86	4.21	0	0.54	3.99	17.85	3.2	3.54
Large Prostate 42	73.85	54.78	15.27	15.35	8.65	8	0.6	1.1	6.15	36.34	8.19	7.75
Large Prostate 43	N/A	N/A	N/A	N/A	N/A	N/A	N/A	N/A	N/A	N/A	N/A	N/A
Large Prostate 44	65.2	43.8	20.3	15.27	18.06	8.5	0.25	0	1.74	28.25	18.2	8.38
Large Prostate 45	N/A	N/A	N/A	N/A	N/A	N/A	N/A	N/A	N/A	N/A	N/A	N/A
Large Prostate 46	67.18	50.6	15.8	13.13	17.35	8.5	0.95	0.45	3.18	30.05	12.5	7.85
Large Prostate 47	76.26	38.85	24.13	5.29	15.73	3.3	0.16	3.01	4.47	26.62	11.69	1.3
Large Prostate 48	58.69	35.22	19.45	30.67	8.17	10.05	0.71	0.79	16.2	28.51	13.66	8.1
Large Prostate 49	74.75	45.6	25	14.7	8.25	7	0.1	1.2	12.1	33.95	17.35	6.86
Large Prostate 50	60.04	48.66	8.52	21.34	14.67	6.1	1.14	0.98	3.7	24.14	6.42	3.3
Large Prostate 51	N/A	N/A	N/A	N/A	N/A	N/A	N/A	N/A	N/A	N/A	N/A	N/A
Large Prostate 52	66.15	39.45	23.45	22	9.65	11.28	0.21	0.55	16.35	24.1	14.15	11.23
Large Prostate 53	57.1	31	26.6	26.1	13.5	6.45	0	0.25	7.5	20.7	13.35	5.73
Large Prostate 54	68.9	35.85	31.15	15.4	14.7	7.6	0.16	0.4	4.05	21.35	13.5	7.48
Large Prostate 55	78	46.85	31	10.65	9.2	6.94	0.79	0.8	19.6	35.9	14.75	6
Large Prostate 56	82.08	50.2	23.74	4.24	11.73	3.3	0.01	2.78	2.14	30.19	14.81	2.4
Large Prostate 57	70.16	42.6	25.45	17.68	10.37	5.99	1.96	1.65	2.2	20.18	7.94	2.92
Large Prostate 58	78.3	59.05	13.6	10.2	8.8	7.11	0.25	0.95	2.5	28.35	8.65	6.81
Large Prostate 59	N/A	N/A	N/A	N/A	N/A	N/A	N/A	N/A	N/A	N/A	N/A	N/A
Large Prostate 60	57.09	33.2	18.4	29.43	12.16	7.5	0.27	1.43	3.3	19.95	11.65	7.17
Large Prostate 61	53.75	41	9.75	8.95	34.45	8.05	0.51	0.8	4.55	15.2	5	6.54
Large Prostate 62	75.85	62.6	11.15	8.4	12.7	8.59	0.45	0.8	5.35	29.85	7.7	7.99
Large Prostate 63	N/A	N/A	N/A	N/A	N/A	N/A	N/A	N/A	N/A	N/A	N/A	N/A
Large Prostate 64	66.4	41.2	25.1	21	9.7	7.4	0.5	0.4	6.1	19.2	12.75	6.83
Large Prostate 65	71.07	49.93	20.81	13.98	12.91	2.84	0.1	1.6	4.56	39.37	16.51	2.55
Large Prostate 66	79.18	52.33	23.91	6.34	12.5	1.8	0.1	6.53	13.51	38	10.05	1.3
Large Prostate 67	58.1	42.6	11.45	33.85	3.75	6.81	0.1	1.1	5.05	27.8	4.45	6.49
Large Prostate 68	60.09	41.55	11.1	23.21	14.99	6.14	1.8	0.24	0.78	22.55	6.35	5.78
Large Prostate 69	69.1	41.77	14.87	10.2	18.55	2.6	0.66	1.75	9.1	22.84	5.98	2.07
Large Prostate 70	63.7	38.15	15.4	22.3	11.3	6.95	0.5	1.75	3.25	19.2	12.1	5.43
Large Prostate 71	65.8	48.6	12.85	13.05	17.7	6.28	0.78	0.9	5.2	28.85	11.1	5.35
Large Prostate 72	38	19.4	18.6	53.7	6.1	9.28	0.27	1.55	1.8	14.95	10.75	5.6
Large Prostate 73	67.55	44.55	18.95	8.75	19.55	11	0.26	1.05	8.35	27.25	13.4	10.59
Large Prostate 74	69.1	44.62	20.21	9.2	18.15	7.68	0.1	1.15	3.3	27.61	20	7.22
Large Prostate 75	61.3	42.55	18.7	27.6	10.85	8.75	0.9	1.45	9.3	26.13	9.38	7.82
Large Prostate 76	71.05	46.8	22	9.65	16.95	7	0.1	2.05	9.4	27.46	9.59	6.69
Large Prostate 77	38	19.4	18.6	53.7	6.1	9.28	0.27	1.55	1.8	14.95	10.75	5.6
Large Prostate 78	56.73	35.55	17.52	10.06	30.21	9.21	1.58	0.55	4.84	18.35	9.26	8
Large Prostate 79	65.86	32	14.45	24.51	83.5	7	0.35	0.93	4.57	14.6	5.45	6.58
Large Prostate 80	70.29	42.85	24.7	14.49	14.3	7	0.81	0.91	11.27	27.23	8.03	6.06

Table S2. Anti-rejection drugs and flow cytometry detection of peripheral blood immune cells in organ transplantation patients.

Transplant Patient	Prostate Volume (mL)	The Use of Anti-rejection Drugs							Peripheral Blood Immune Cell Flow Cytometry Detection										
		Cyclosporine	Rapamycin	FK506	Mizoribine	Mycophenolate mofetil	Prednisone	Methyl-prednisolone	CD3+CD4+CD8-	CD3+CD4-CD8+	CD3-CD(16+56)+	CD3-CD19+	CD4+CD25+/CD4+	CD8+CD25+/CD8+	CD3+CD69+	CD3+HLA-DR+	CD4+CD29+	CD8+CD28+	Number of CD4+CD25+CD127-
1	10.36	✓				✓		✓	44.6	17.55	27.75	2.12	3.14	2.56	1.18	2.86	28.7	7.94	2.91
2	11.95			✓		✓		✓	34.2	25.53	15.5	9.3	7.32	0.75	6.3	11.8	20.4	10.8	5.63
3	13.10		✓			✓		✓	49.6	29.5	12.5	6.9	4.64	0.32	1.5	4.2	40.2	12.7	3.83
4	27.23			✓		✓		✓	45.55	31.65	8.17	2.22	6.92	0.47	1.46	4.2	33.1	13.45	4.06
5	17.31	✓				✓	✓		69.6	16.73	5.87	6.41	5.41	0.59	2.83	4.67	32.57	6.91	4.84
6	33.67		✓			✓		✓	29.87	30.84	29.88	1.74	7.25	0.7	6.87	5.67	17.19	18.54	5.43
7	16.05		✓			✓		✓	36.74	37.84	12.5	0.88	3.29	0.87	6.03	1.32	29.35	8.81	1.2
8	14.55			✓	✓			✓	31.53	17.42	32.5	11.5	7.21	2.72	1	6.5	24.74	6.83	3.3
9	28.17			✓		✓	✓		40.1	27.84	13.3	14.2	6.43	2.96	3.1	3	22.35	17.21	3.34
10	25.83			✓		✓		✓	58.09	14.86	17.1	6.4	4.59	3.45	2.9	0.6	31.28	7.08	2.29
11	17.19		✓			✓		✓	26.15	44	14.67	2.82	4.02	0	21.28	17.05	18.95	3.9	2.86
12	10.33	✓				✓		✓	38.84	34.39	18.28	4.48	5.45	0.62	9.78	12.43	30.32	12.15	3.27
13	22.51		✓	✓		✓		✓	36.24	31.03	17.99	3.11	4.52	0.32	4.5	4.64	23.14	8.04	4.24
14	24.50	✓				✓	✓		36.62	40.92	15.93	5.21	7.7	0.68	1.98	5.52	25.65	10.57	4
15	19.80	✓				✓		✓	63.3	18.1	12.8	3	3.7	0.2	0.8	3.5	30.3	11.5	N/A
16	22.34			✓		✓	✓		24.2	39	15.08	6.73	2.48	0.26	8.24	5.83	18.78	11.45	2.09
17	36.56			✓		✓		✓	33.37	15.4	44	3.6	3.55	0.62	0.2	2.7	28	8.3	2.85
18	18.82	✓				✓		✓	46.93	27.83	9.83	9.01	4.7	1.18	1.64	4.51	31.5	9.75	4.4
19	20.76			✓		✓		✓	39.44	21.72	29.26	2.76	2.63	1.1	1.56	4.72	22.4	13	1.72
20	15.44	✓			✓		✓		37.02	49.63	4.99	0.71	6.51	0.39	1.48	3.43	30.71	9.37	3.08
21	33.94		✓			✓		✓	53.59	19.54	2.93	6.77	11.32	0.58	51.81	9.22	25.65	7.78	N/A
22	16.05		✓			✓		✓	17	27.69	45.91	2.16	3.25	0.25	1.58	4.91	12.79	2.95	3.07
23	24.89			✓		✓		✓	36.65	19.55	20	17.1	6.41	0.55	2.2	4.1	18.8	10.1	4.08
24	22.46			✓		✓		✓	35.4	22.5	28	6.1	8.19	1.78	1.6	5.6	23.5	8.9	5.08
25	20.63	✓	✓					✓	34.1	40.5	12.8	3.1	3.5	0.1	5.4	4.1	24.8	6.6	N/A
26	11.68	✓				✓		✓	38.74	30.06	11.93	1.41	4.8	0.34	8.57	4.99	17.26	9.51	3.47
27	25.81			✓		✓		✓	43.55	34.7	3.44	3.18	6.5	0.43	2.62	0.41	28.27	6.78	6.39
28	27.23			✓		✓		✓	20.8	33.03	35.66	2.81	6.6	0.31	2.02	9.04	9.03	4.62	5
29	9.75			✓		✓		✓	39.53	35.34	10.5	11.9	2.45	0	1.6	2.1	25.63	5.05	1.63
30	13.25	✓				✓		✓	N/A	N/A	N/A	N/A	N/A	N/A	N/A	N/A	N/A	N/A	N/A
31	18.72			✓		✓		✓	N/A	N/A	N/A	N/A	N/A	N/A	N/A	N/A	N/A	N/A	N/A
32	20.66		✓			✓		✓	N/A	N/A	N/A	N/A	N/A	N/A	N/A	N/A	N/A	N/A	N/A
33	26.25			✓		✓		✓	N/A	N/A	N/A	N/A	N/A	N/A	N/A	N/A	N/A	N/A	N/A
34	21.30			✓		✓		✓	N/A	N/A	N/A	N/A	N/A	N/A	N/A	N/A	N/A	N/A	N/A
35	16.82	✓				✓		✓	N/A	N/A	N/A	N/A	N/A	N/A	N/A	N/A	N/A	N/A	N/A
36	34.07		✓			✓	✓		N/A	N/A	N/A	N/A	N/A	N/A	N/A	N/A	N/A	N/A	N/A
37	14.11			✓		✓		✓	N/A	N/A	N/A	N/A	N/A	N/A	N/A	N/A	N/A	N/A	N/A
38	12.97			✓	✓			✓	N/A	N/A	N/A	N/A	N/A	N/A	N/A	N/A	N/A	N/A	N/A
39	30.30		✓			✓		✓	N/A	N/A	N/A	N/A	N/A	N/A	N/A	N/A	N/A	N/A	N/A
40	21.28		✓			✓		✓	N/A	N/A	N/A	N/A	N/A	N/A	N/A	N/A	N/A	N/A	N/A
41	21.15			✓		✓		✓	N/A	N/A	N/A	N/A	N/A	N/A	N/A	N/A	N/A	N/A	N/A
42	35.27			✓		✓		✓	N/A	N/A	N/A	N/A	N/A	N/A	N/A	N/A	N/A	N/A	N/A
43	22.63		✓			✓		✓	N/A	N/A	N/A	N/A	N/A	N/A	N/A	N/A	N/A	N/A	N/A
44	15.12			✓		✓		✓	N/A	N/A	N/A	N/A	N/A	N/A	N/A	N/A	N/A	N/A	N/A
45	21.67		✓			✓		✓	N/A	N/A	N/A	N/A	N/A	N/A	N/A	N/A	N/A	N/A	N/A
46	6.18	✓	✓					✓	N/A	N/A	N/A	N/A	N/A	N/A	N/A	N/A	N/A	N/A	N/A
47	25.83		✓			✓		✓	N/A	N/A	N/A	N/A	N/A	N/A	N/A	N/A	N/A	N/A	N/A
48	34.15			✓		✓		✓	N/A	N/A	N/A	N/A	N/A	N/A	N/A	N/A	N/A	N/A	N/A
49	14.41			✓		✓		✓	N/A	N/A	N/A	N/A	N/A	N/A	N/A	N/A	N/A	N/A	N/A
50	21.50			✓		✓		✓	N/A	N/A	N/A	N/A	N/A	N/A	N/A	N/A	N/A	N/A	N/A
51	13.37			✓		✓	✓		N/A	N/A	N/A	N/A	N/A	N/A	N/A	N/A	N/A	N/A	N/A
52	15.16	✓	✓			✓		✓	N/A	N/A	N/A	N/A	N/A	N/A	N/A	N/A	N/A	N/A	N/A
53	36.56			✓	✓			✓	N/A	N/A	N/A	N/A	N/A	N/A	N/A	N/A	N/A	N/A	N/A
54	36.56		✓			✓		✓	N/A	N/A	N/A	N/A	N/A	N/A	N/A	N/A	N/A	N/A	N/A
55	22.96			✓		✓		✓	N/A	N/A	N/A	N/A	N/A	N/A	N/A	N/A	N/A	N/A	N/A
56	14.98		✓			✓	✓		N/A	N/A	N/A	N/A	N/A	N/A	N/A	N/A	N/A	N/A	N/A
57	14.51			✓		✓		✓	N/A	N/A	N/A	N/A	N/A	N/A	N/A	N/A	N/A	N/A	N/A
58	20.03			✓		✓	✓		N/A	N/A	N/A	N/A	N/A	N/A	N/A	N/A	N/A	N/A	N/A
59	24.16	✓				✓		✓	N/A	N/A	N/A	N/A	N/A	N/A	N/A	N/A	N/A	N/A	N/A
60	22.93	✓				✓		✓	N/A	N/A	N/A	N/A	N/A	N/A	N/A	N/A	N/A	N/A	N/A
61	17.89	N/A	N/A	N/A	N/A	N/A	N/A	N/A	N/A	N/A	N/A	N/A	N/A	N/A	N/A	N/A	N/A	N/A	N/A
62	14.15	N/A	N/A	N/A	N/A	N/A	N/A	N/A	N/A	N/A	N/A	N/A	N/A	N/A	N/A	N/A	N/A	N/A	N/A
63	22.34	N/A	N/A	N/A	N/A	N/A	N/A	N/A	N/A	N/A	N/A	N/A	N/A	N/A	N/A	N/A	N/A	N/A	N/A
64	27.71	N/A	N/A	N/A	N/A	N/A	N/A	N/A	N/A	N/A	N/A	N/A	N/A	N/A	N/A	N/A	N/A	N/A	N/A
65	17.64	N/A	N/A	N/A	N/A	N/A	N/A	N/A	N/A	N/A	N/A	N/A	N/A	N/A	N/A	N/A	N/A	N/A	N/A
66	26.25	N/A	N/A	N/A	N/A	N/A	N/A	N/A	N/A	N/A	N/A	N/A	N/A	N/A	N/A	N/A	N/A	N/A	N/A
67	8.79	N/A	N/A	N/A	N/A	N/A	N/A	N/A	N/A	N/A	N/A	N/A	N/A	N/A	N/A	N/A	N/A	N/A	N/A

Abbreviations

BPH: Benign prostatic hyperplasia; LUTS: lower urinary tract symptoms; Treg: Regulatory T cell; AID: autoimmune diseases; mTOR: mammalian target of rapamycin; scRNA-seq: single-cell RNA sequencing; ThuLEP: thulium laser enucleation of the prostate; PCA: principal component analysis; DEGs: Differentially expressed genes; GSEA: Gene Set Enrichment Analysis; OR: Odds ratio; KEGG: Kyoto Encyclopedia of Genes and Genomes; GO: Gene Ontology; GZMB: granzyme B; GZMK: granzyme K; GZMA: granzyme A; CNIs: calcineurin inhibitors; CsA: cyclosporine A; MMF: mycophenolate mofetil; TCR: T-cell receptor; SLE: systemic lupus erythematosus; 5ARIs: 5α-Reductase Inhibitors; NSAIDs: nonsteroidal anti-inflammatory drugs.
